# Multiscale characteristics and cracking behavior in reservoir sandstone under dry-wet cycles: Insights from NMR, AE and SEM

**DOI:** 10.1038/s41598-026-46226-1

**Published:** 2026-04-01

**Authors:** Pei He, Ruide Lei, Pengcheng Zhao, Ye Zhang, Linsen Zhou, Dong Wang

**Affiliations:** 1 Key Laboratory of Shale Gas Exploration, Chongqing Institute of Geology and Mineral Resources, Ministry of Natural Resources, Chongqing, 401120 China; 2 National and Local Joint Engineering Research Center of Shale Gas Exploration and Development, Chongqing Institute of Geology and Mineral Resources, Chongqing, 401120 China; 3 College of Civil Engineering, Sichuan University of Science and Engineering, Zigong, 643000 China

**Keywords:** Dry-wet cycles, Multiscale evolution, Failure precursor, Acoustic emission, Nuclear magnetic resonance, Energy science and technology, Engineering, Natural hazards, Solid Earth sciences

## Abstract

The periodic water-level fluctuations in the hydro-fluctuation belt of the Three Gorges reservoir progressively degrade the mechanical integrity of reservoir rock mass, thereby promoting slope instability and related geohazards. To elucidate the multiscale damage evolution and cracking behavior of sandstone under hydromechanical loading, here we conducted a series of dry-wet cyclic experiments, synchronized with acoustic emission (AE), nuclear magnetic resonance (NMR), and scanning electron microscopy (SEM). The results show that the early warning points occur at 91.76%-98.53% of peak stress, whereas the critical fracture point consistently appears beyond 99% of peak stress. When the number of dry-wet cycle increases from 0 to 30, the proportion of tensile cracks increases from 31.05% to 48.93%, indicating a progressive transition from shear-dominated failure to a tensile-shear mixed mode. NMR results reveal an exponential increasement in porosity with the number of dry-wet cycles. The *T*_2_ spectrum evolves from unimodal to bimodal, suggesting enhanced the pore-scale heterogeneity. This phenomenon is accompanied by a significant reorganization of the pore structure, with the proportion of micropores decreasing from 8.09% to 0.24% and that of macropores increasing from 50.78% to 65.30%. SEM observations corroborate the multiscale degradation mechanism, revealing that early-stage damage is characterized by dissolution pits and isolated microcracks on mineral surfaces. In contrast, progressive cycling substantially weakens interparticle cementation, generates abundant debris, and promotes the coalescence of microcracks into macroscopic fractures. This integrated AE-NMR-SEM framework links pore-scale evolution to macroscopic failure processes, providing a quantitative method for precursor identification and failure-mode transition assessment in reservoir rock mass.

## Introduction

**Fig. 1:**
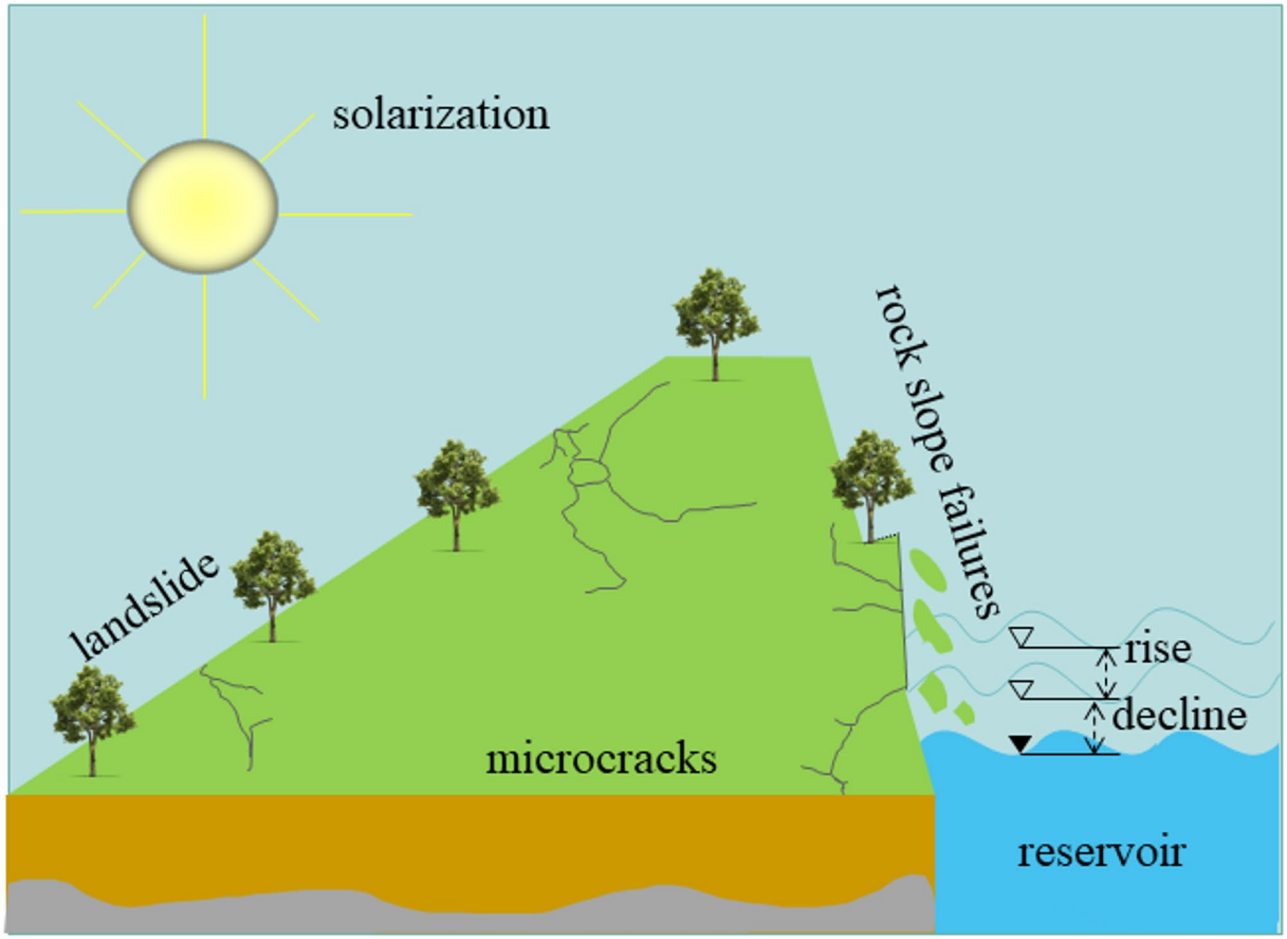
Schematic diagram of geological hazards induced by cyclic drying-wetting.

Rock masses in the hydro-fluctuation belt of the Three Gorges reservoir area are frequently encountered by dry-wet cycling environment. Such environmental stress not only alters the geological environment of the reservoir area, but also leads to the progressive deterioration of the mechanical properties of the rock mass. Specifically, these cycles facilitate the connectivity of microcracks, thereby diminishing the integrity of the rock mass, and may eventually trigger geological disasters such as slope failures, landslides, and collapses as shown in Fig. 1^1^. Under the combined action of external loads and pore water pressure on rock mass, damage will occur at the mineral interface and gradually extend to large-scale degradation^2^. Also, the rocks are mainly composed of various minerals such as quartz, feldspar, and mica. These components exhibit obvious heterogeneity and anisotropy, which makes it difficult to accurately capture the damage evolution within the rock mass. Therefore, a thorough understanding of the microscopic degradation mechanism and damage evolution under such cyclic hydromechanical loading is paramount to accurately assessing rock durability.

Extensive studies have increasingly focused on the mesoscale degradation of rocks subjected to dry-wet cycling. Xie et al.^3^, examined damage behavior of sandstone under wet-dry cycles using low-field nuclear magnetic resonance (NMR). The results indicate a continuous increase in the number of pores, accompanied by a gradual enlargement of pore size. With further development, interconnected microcracks form and propagate. These observations demonstrate that dry-wet cycles promote the transition of damage from pore expansion to crack development. Song^4^, investigated the internal changes of weakly cemented materials under different dry-wet conditions. The results show that the *T*_2_spectrum shifts to the right as the number of cycles increases, indicating sustained enlargement of pore size. Meanwhile, the degree of damage continues to accumulate, whereas the growth rate gradually decreases. This trend suggests that structural degradation induced by dry-wet cycling is more pronounced at early stages and becomes less severe with continued cycling. In addition, Wang et al.^5^, analyzed the pore structure of sandstone under cyclic conditions. The results show that the diffusion behavior of pores mainly depends on pore size and the degree of damage with increasing cycle number. The most significant reduction occurs in the diffusion capacity of micropores, followed by intermediate pores, and finally macropores. Also, Zeng et al.^6^, explored the effect of cyclic humidity on salt rock and examined the evolution of its pore structure during repeated drying and wetting cycles. They found that fluid viscosity within mesopores hindered brine replenishment during initial cycles, prolonged cycling activated self-healing mechanisms in macropores, suggesting a complex competition between damage and recovery within the rock matrix. The aforementioned studies have deepened the understanding of pore-scale degradation in rocks under cyclic dry-wet conditions, particularly through NMR characterization of pore evolution and microstructural alteration. Despite NMR provides valuable insights into the dynamic behavior of internal pore networks, its spatial resolution remains limited for visualizing microcrack morphology and localized damage zones.

To address this limitation, modern microscopic analysis techniques have been increasingly employed to identify and characterize damage at the microstructural level^7^. Zhou^8^, conducted scanning electron microscope (SEM) observations on sandstone subjected to 10 cycles, and obtained the characteristics of microscopic morphology. The results show that clay minerals undergo non-uniform expansion under dry-wet cycling. This process induces the initiation and propagation of cracks within mineral particles. Yang^9^, selected basalt as the research material and performed a detailed analysis of the dissolution behavior of mineral particles under dry-wet cycling using SEM. The results indicate progressive development of crystal cracks with increasing number of cycles. After 40 dry-wet cycles, evident crack propagation and pore dissolution are observed. Yu^10^, investigated the multiscale crack behavior of silty mudstone using SEM. Due to mineral dissolution or physical erosion, fracture roughness and fractal dimension increase with the cyclic number. Under identical cycling conditions, higher roughness is observed at a cycle amplitude of 10%. Kou et al.^11^, demonstrated crack morphology under varying fluid pressures using SEM. The results show that the roughness of the microcrack decreased with increasing pressurized water, elucidating the mechanism of hydraulic-induced microdamage in precracked specimens. Zhao^12^, investigated the microstructure of metamorphic sandstone through cyclic water-rock interaction tests, identifying mineral hydration, dissolution, and pore expansion as the dominant factors driving damage progression. Despite significant advances in microscopic and mesoscopic characterization, current studies on rock degradation often remain confined to isolated scales, with limited integration across physical, chemical, and mechanical processes. As a result, most research lacks a unified multiscale experimental framework to link microscale mechanisms with macroscale mechanical behavior. Under external loading, the manner in which pre-existing microstructural damage caused by wet-dry cycling dominates subsequent damage and failure remains insufficiently understood. Acoustic emission (AE) technology, as a non-destructive monitoring approach, has been widely used in rock mechanics. (Huang^13^, Liu^14^, Long^15^, Tan^16^,; Zhao et al., 2026) This method records the transient elastic energy released by the initiation, propagation, and coalescence of microcracks within rocks. It enables real-time capture of microcrack activity during mechanical loading of rocks. Through detailed analysis of AE events, the damage evolution of pre-damaged rock materials under load can be characterized.

The present study adopts a synergistic approach that combines AE, NMR, and SEM to investigate the multiscale degradation mechanisms of sandstone under varying cyclic dry-wet conditions. This provides insight into the micro- and macro-scale damage and failure mechanisms of sandstone under hydro-mechanical coupling. Firstly, the characteristics of AE energy during the entire loading process were analyzed. By using the ratio of RA (rise time/peak amplitude) and AF (ring counts/duration), the evolution of tensile and shear microcracks was quantitatively characterized under different loading stages. In addition, the theory of critical slowing down was introduced to analyze precursory information prior to failure. At the mesoscopic scale, NMR was used to quantify the pore structure of sandstone subjected to different dry-wet cycles. Also, the *T*_2_ spectrum distribution, porosity, pore-size distribution, and the segmented fractal dimension of sandstone were primarily investigated in detail.

## Experimental methodology

### Sample preparation

**Fig. 2:**
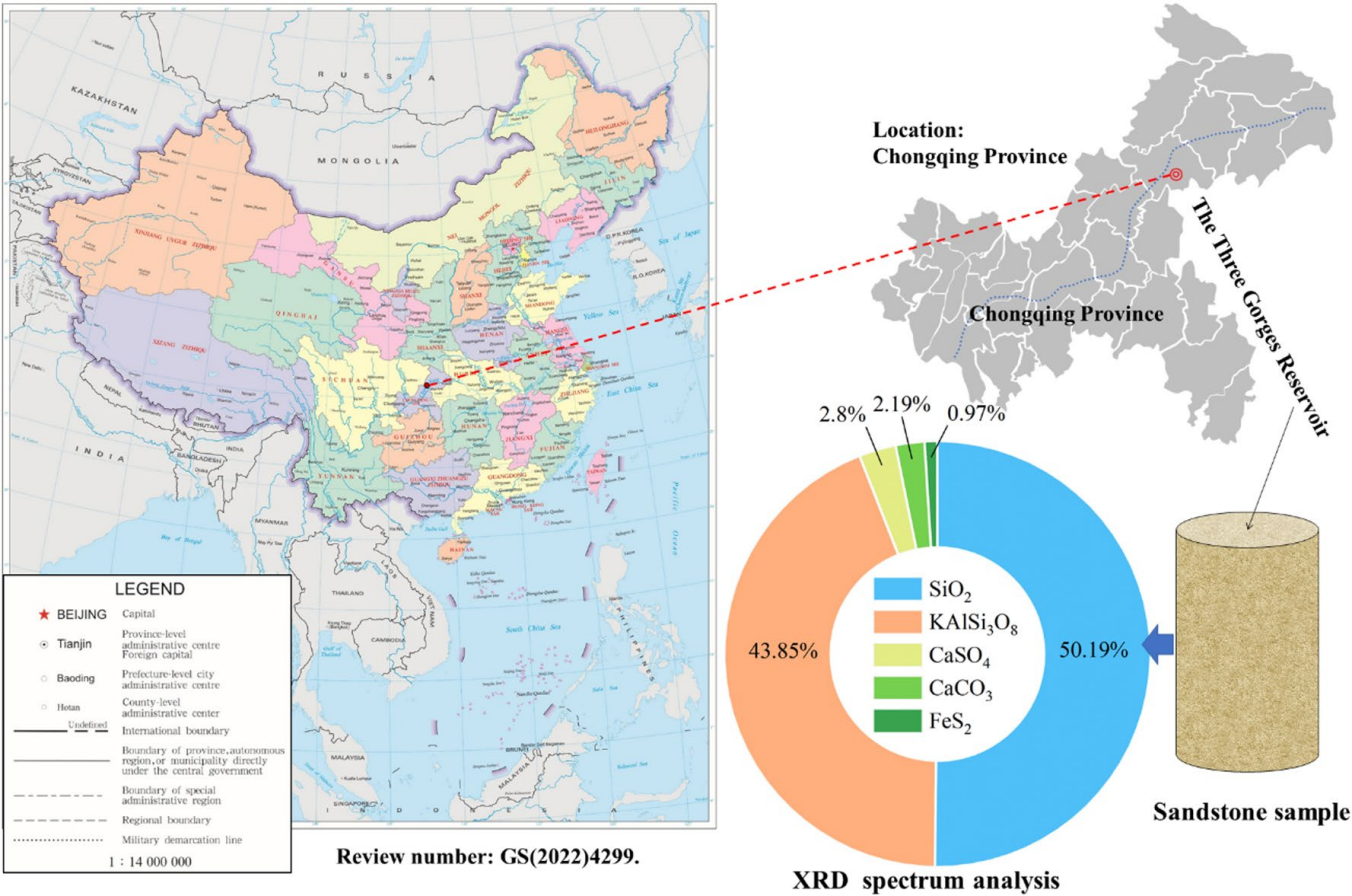
The sample location and mineral composition of sandstone.

The sandstone specimens utilized in this study were obtained from the Ecological Buffer Zone of the Three Gorges Reservoir area, as shown in Fig. 2. The samples appear gray-white under natural conditions, and the surfaces show no visible cracks. The main components of the tested sandstone include quartz, feldspar, and clay minerals. To minimize variability, all test specimens were extracted from a single homogenous parent rock block. Specimen preparation strictly adhered to the recommended guidelines of the International Society for Rock Mechanics (ISRM). Cylindrical specimens, with a diameter of 25 mm and a height of 50 mm, were drilled perpendicular to the bedding plane, maintaining stringent tolerances for surface unevenness and perpendicularity deviation. At the same time, at least three specimens with dimensions of 5 mm × 5 mm × 3 mm were prepared for SEM analysis. After that, the longitudinal wave velocity of each specimen was measured to assess homogeneity. To ensure representative and comparable observations across different cyclic conditions, a systematic sampling strategy was employed. For each specimen, at least five randomly selected fields of view were imaged at multiple magnifications (500×, 1000×, 2000×, and 5000×). For specimens that developed visible fractures, observations were conducted on both the fracture surfaces and matrix regions located at least 2 mm away from any visible fracture.

### Testing instruments

**Fig. 3:**
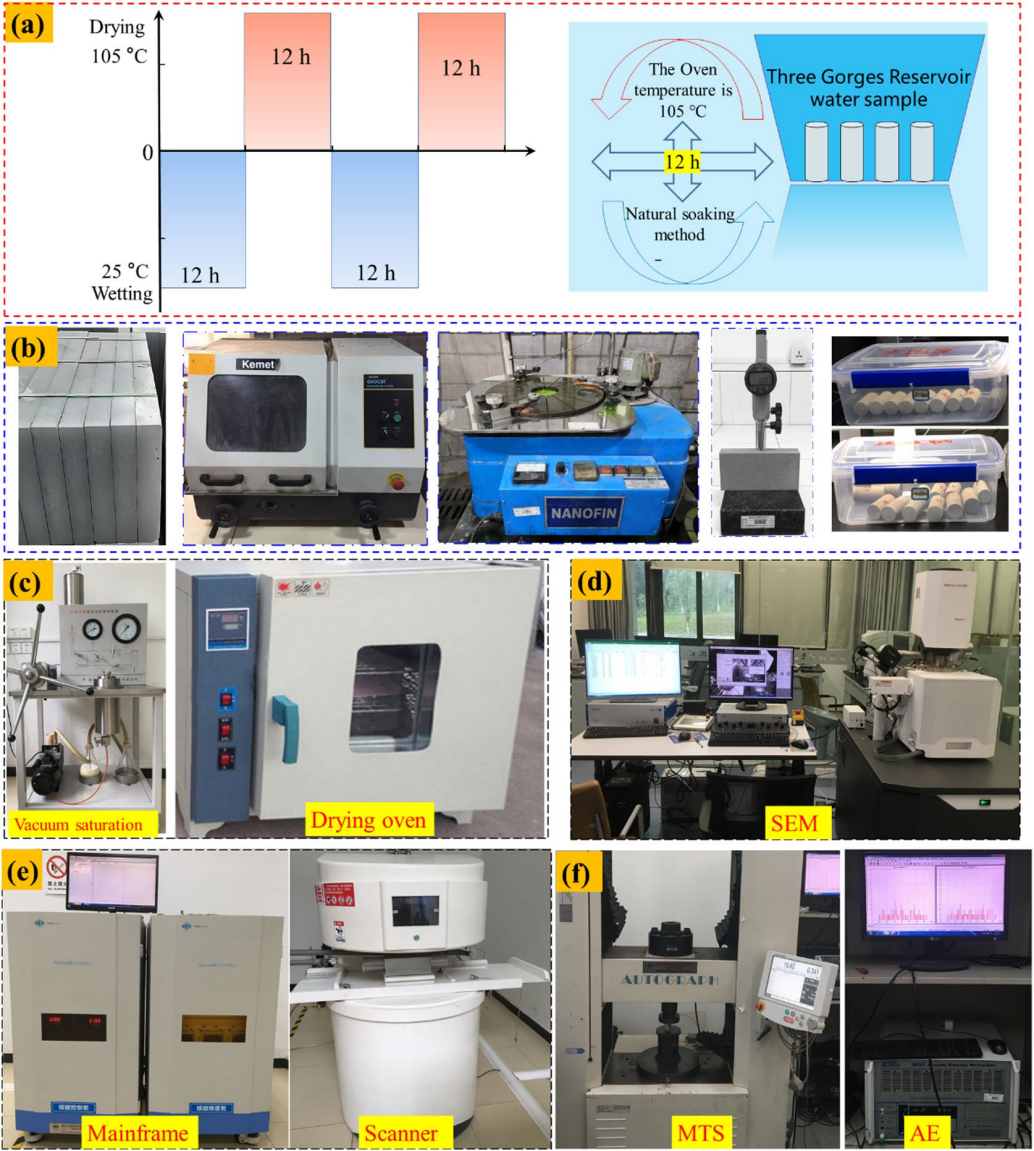
Experimental methods and procedure: (**a**) schematic diagram of dry-wet path, (**b**) sandstone processing, (**c**) drying oven device, (**d**) SEM tests, (**e**) NMR tests, and (**f**) MTS and AE testing system.

To replicate the environmental conditions of the Ecological Buffer Zone of the Three Gorges Reservoir, the sandstone specimens were subjected to controlled drying-wetting cycle experiments. The saturated specimens were then divided into five groups for the cyclic tests. Each drying-wetting cycle comprised a 12-hour immersion period to achieve saturation, followed by a 12-hour drying phase in an oven at 105 °C. Subsequently, the samples were cooled to ambient temperature, thus completing a single cycle. To ensure complete saturation of the tested specimens, the saturation was implemented before each NMR test. Specimens were first placed in a vacuum chamber at −0.1 MPa for 4 h to evacuate air from the pore space. Subsequently, distilled water was introduced while maintaining the vacuum, and the specimens were immersed for 12 h. Finally, saturation was verified by weighing specimens at 4 h intervals until mass stabilization. The five groups of specimens were subjected to 0, 5, 10, 20, and 30 cycles, respectively. A schematic of the wetting-drying cycle procedure is presented in Fig. 3(a).

The experimental apparatus utilized in this study included an NMR testing system, an SEM system, an ISTRON testing device, and an AE monitoring system, as depicted in Figs. 3d-f. The instrument featured a maximum accelerating voltage of 200 kV and a point resolution of 0.25 nm in TEM mode. To investigate the deterioration mechanism at the pore scale, NMR technology was applied using a system consisting of a magnet, probe, and main cabinet, with a magnetic field strength of 0.3 ± 0.05 T, an operating temperature of 32 °C, and a radio frequency range of 2–30 MHz. The mechanical testing was conducted using an ISTRON electro-hydraulic servo-controlled system with a maximum axial load capacity of 250 kN. The force and displacement measurement accuracies are 0.5% and 0.1%, respectively. A constant displacement loading mode was applied at a rate of 0.05 mm/min. Concurrently, AE signals were collected by the PCI-II system, which was configured with an acquisition threshold of 40 dB, a gain of 60 dB, and a sampling frequency of 1 MHz.

## Experimental results

### Macrostructural characteristics of sandstone under different dry-wet cycles

#### AE characteristics

**Fig. 4:**
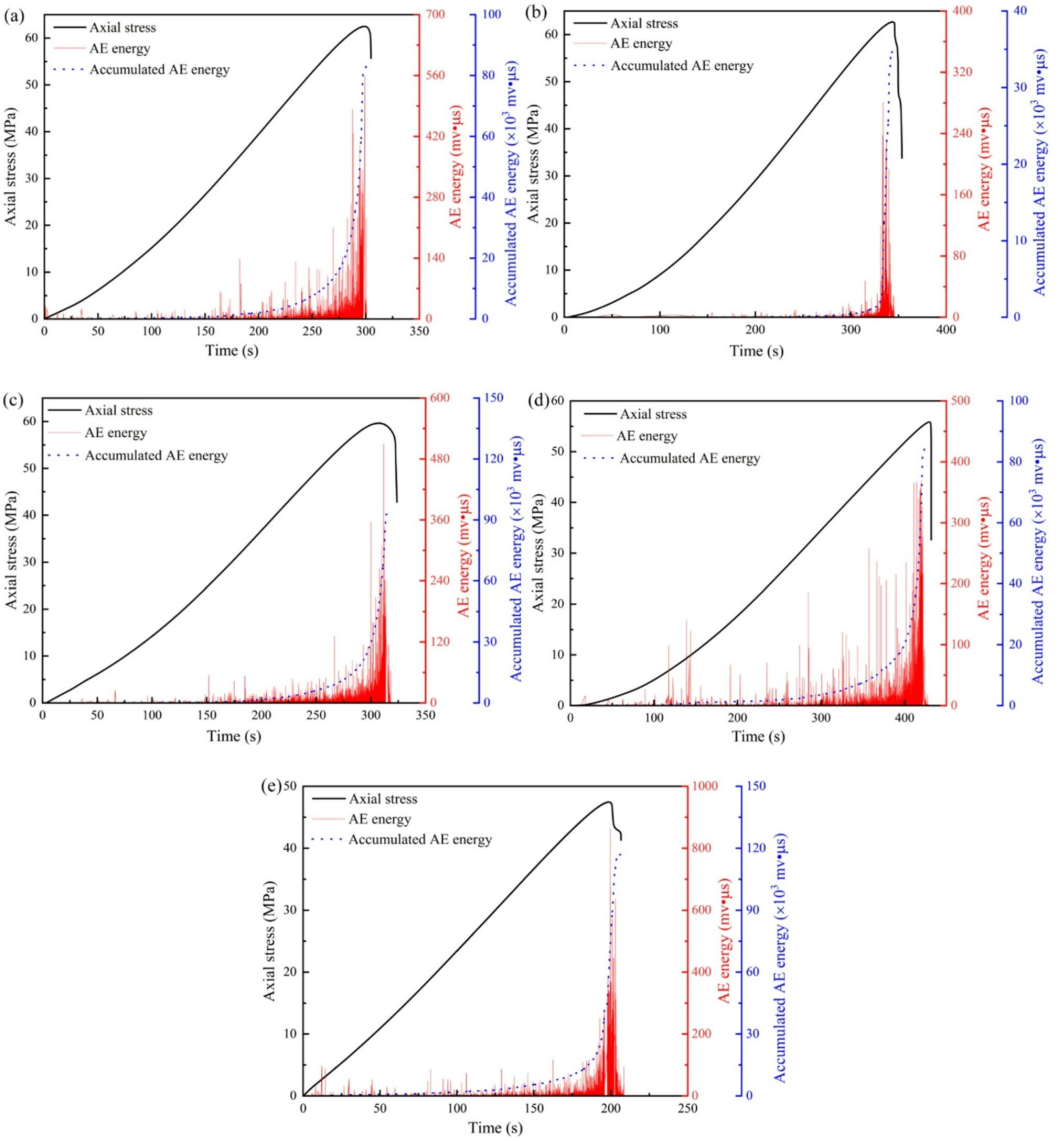
The evolution of AE energy for sandstone under different dry-wet cycles: (**a**) *N* = 0, (**b**) *N* = 5, (**c**) *N* = 10, (**d**) *N* = 20, and (**e**) *N* = 30.

Figure 4 shows the temporal evolution of axial stress, AE energy, and cumulative AE energy for sandstone subjected to varying dry-wet cycles. In general, the cumulative AE energy curve exhibits a steadily increasing trend, whereas the instantaneous AE energy is characterized by a multi-peak episodic pattern. The entire loading process can be divided into four distinct stages based on AE characteristics: compaction, linear elasticity, crack propagation, and post-peak.

For the untreated specimen (Fig. 4a), AE energy remains relatively low during the initial compaction stage, with only sporadic events detected. The cumulative AE energy exhibits a gradual and steady rise, indicating few initial damage. After 5 cycles (Fig. 4b), a noticeable increase in AE activity is observed during compaction stage, suggesting that initial damage (microcracks and pores) has been enhanced by the cyclic treatment. The cumulative energy curve shows a slightly steeper slope compared to Fig. 4a, reflecting accelerated damage accumulation. The pre‑peak AE energy bursts become more frequent and intense. At 10 cycles (Fig. 4c), AE events during the compaction stage are even more pronounced, with the cumulative energy beginning to rise earlier than in previous cases. The slope of the cumulative energy curve steepens further during the crack propagation stage, and the peak AE energy reaches higher magnitudes, reflecting intensified microcracking activity. After 20 cycles (Fig. 4d), AE activity is detected almost from the beginning of loading, with continuous energy releases throughout the compaction and elastic stages. The cumulative energy curve shows a nearly exponential increase, marked by multiple sharp rises corresponding to clusters of high‑energy AE events. Sustained AE activity in the post‑peak region exhibits more complex fracture development. The most dramatic changes are observed in the specimen subjected to 30 cycles (Fig. 4e). High‑amplitude AE events occur even at very low stress levels, and the cumulative energy curve rises steeply from the outset. Consequently, the distinction between loading stages becomes blurred as damage accumulates continuously throughout the test. Both the peak AE energy and the cumulative energy reach its maximum values among all specimens, demonstrating that extensive pre‑existing damage significantly accelerates and intensifies the fracture process.

#### Precursory characteristics

Critical slowing down, a concept originating from statistical physics, characterizes the behavior of a system approaching a critical transition point. During a phase shift from one stable state to another, the system in the vicinity of the critical point exhibits fluctuations with increasing amplitude and duration, a decelerated recovery rate, and reduced resilience to return to its equilibrium. The anticipation of such transitions is made possible by monitoring several statistical indicators of the system’s state^17–19^. Accordingly, the present study employs the variance of AE parameters as the indicators to identify the precursory signals of imminent rock failure.

1 $$D=\frac{1}{S}{\sum\limits_{{{i_1}=1}}^{N} {\left( {{X_{{i_1}}} - \bar {X}} \right)} ^2}$$

where *D* is the variance, *X*_*i*_ is the *i*-th reference point of AE energy, $\overline{X}$ is the mean value of the AE dataset, and *S* is the total number of AE events recorded.

**Fig. 5:**
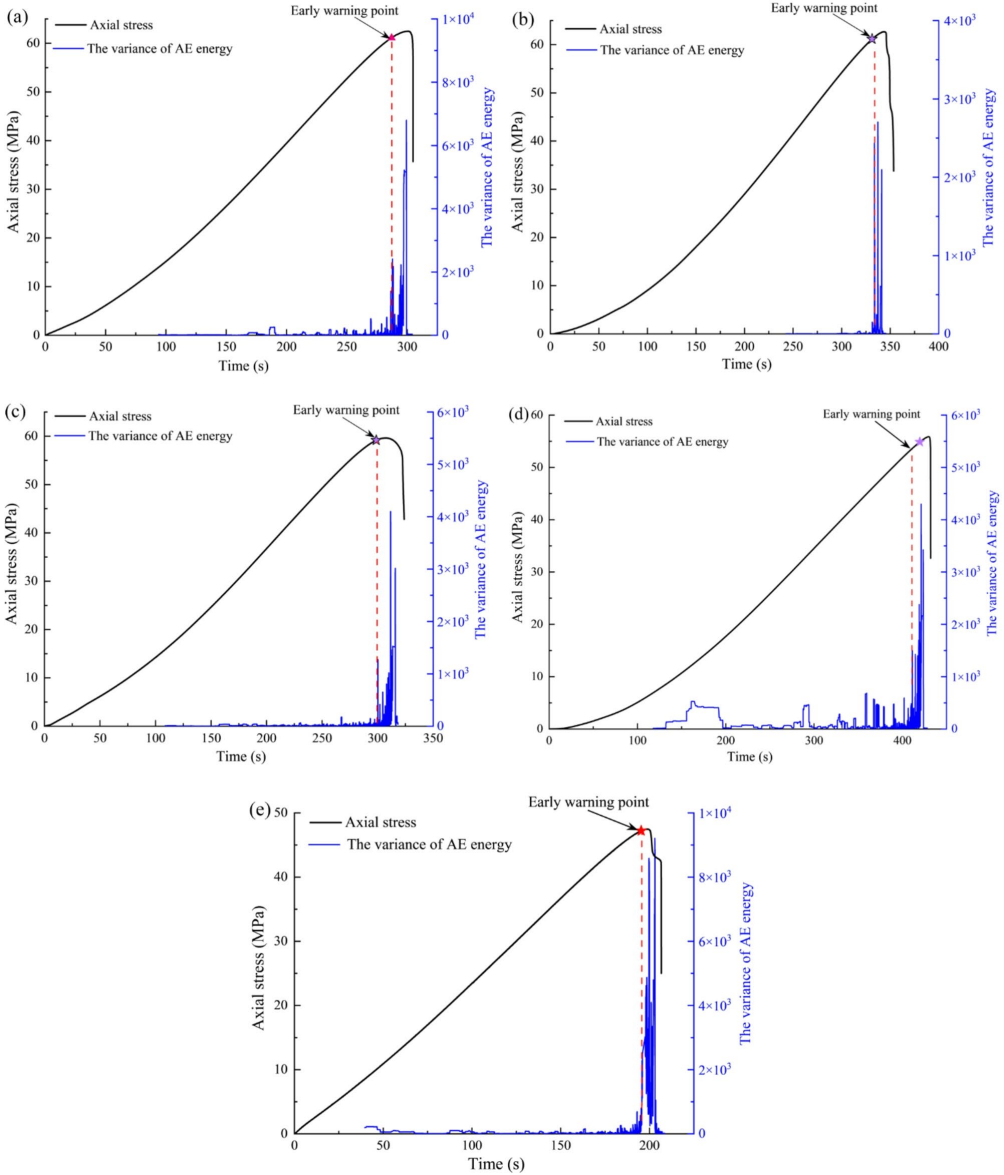
The evolution of the AE energy variance for sandstone under different dry-wet cycles: (**a**) *N* = 0, (**b**) *N* = 5, (**c**) *N* = 10, (**d**) *N* = 20, and (**e**) *N* = 30.

Figure 5 shows the variance of these AE parameters plotted against axial stress over time. The variance curves corresponding to AE count, energy, and rise time all exhibit a similar pattern, characterized by relative stability prior to a critical event. In this study, the initial pronounced increase in AE parameter variance is defined as the early warning point, and the peak variance value is identified as the critical fracture point. Observation from Fig. 5, it can be found that the early warning points range from 91.76% to 98.53% of the peak stress. In addition, the critical fracture points consistently occur at stress levels exceeding 99% of the peak.

For the untreated specimen (Fig. 5a), the AE energy variance remains at a low and stable level during the initial loading stage. The first significant increase occurs at approximately 281 s, which is identified as the early warning point. Subsequently, the variance accelerates sharply, reaching its peak at around 310 s, which corresponds to the critical fracture point. The early warning point occurs at 90.6% of the time to failure, indicating that precursor signals emerge well before final failure. After 5 cycles (Fig. 5b), the early warning point is delayed to approximately 335 s, with the critical fracture point at 350 s. Compared to the untreated specimen, both the early warning and failure times are extended, suggesting that moderate cyclic treatment may temporarily enhance ductility or delay instability. The early warning point corresponds to 95.7% of the failure time. At 10 cycles (Fig. 5c), the early warning point shifts to 310 s, and the critical fracture point occurs at 325 s. After 20 cycles (Fig. 5d), the early warning point corresponds to 97.4% of the failure time. This suggests that while the rock maintains stable crack growth for a longer duration, the transition to instability is extremely rapid once initiated. Conversely, for the specimen subjected to 30 cycles (Fig. 5e), both the early warning point and failure occur much earlier, at 195 s and 204 s, respectively. These findings signify that failure precursors form during the unstable crack propagation stage, characterized by the rapid expansion and coalescence of microcracks into macroscopic fractures. Consequently, a dramatic increase in the variance of these three AE parameters can be adopted as a quantitative criterion for identifying the onset of instability in the rock.

#### Cracking characteristics

In AE analysis, the RA and AF values are fundamental parameters for characterizing the nature of the signal source. RA is defined as the ratio of rise time to amplitude, while AF is the ratio of count to duration time^20^. These parameters collectively describe the dynamics of energy release from the source. Tensile fracture events are typically associated with abrupt energy release, generating AE signals with short rise times and high-frequency components, thereby corresponding to low RA and high AF values. In contrast, shear fracture involves a more protracted energy release, leading to signals with longer rise times and lower frequencies, which manifest as high RA and low AF values^21,22^. Consequently, the analysis of the RA-AF distribution serves as a robust method for discriminating between tensile and shear failure modes, offering crucial insights into the material’s underlying damage mechanism.

2 $$AF=\frac{C}{{{D_T}}}$$

3 $$RA=\frac{{{R_T}}}{A}$$

To more comprehensively reveal the failure mechanism of cracking for sandstone under different loading stages, the RA and AF values were normalized. The normalization equation is expressed as follows:

4 $$x'=\frac{{x - \hbox{min} \left\{ {{x_1},{x_2},{x_3},...,{x_n}} \right\}}}{{\hbox{max} \left\{ {{x_1},{x_2},{x_3},...,{x_n}} \right\} - \hbox{min} \left\{ {{x_1},{x_2},{x_3},...,{x_n}} \right\}}}$$

where $x'$ is the normalized sample value, and *x* is the sample value.

**Fig. 6:**
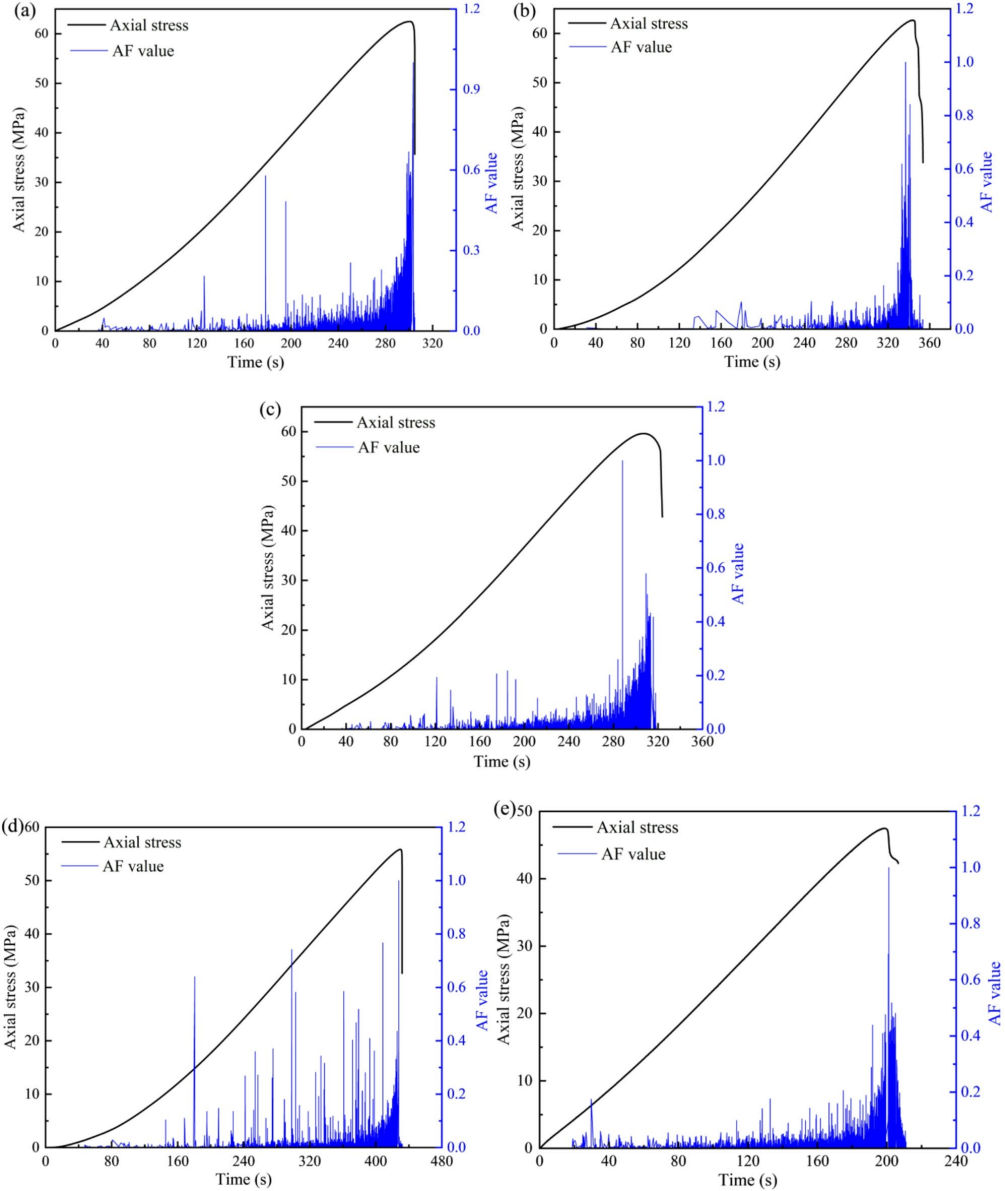
Evolution of the normalized AF values for sandstone under different dry-wet cycles: (**a**) *N* = 0, (**b**) *N* = 5, (**c**) *N* = 10, (d) *N* = 20, and (e) *N* = 30.

Figure 6 shows the temporal evolution of the normalized AF value, and the stress–strain curve. For the intact specimen (Fig. 6a), the AF value maintains a high plateau during the elastic deformation phase, then declines gradually as macroscopic failure approaches. This pattern indicates a transition from the early-stage tensile cracking to shear-dominated failure, aligning with the typical response of relatively brittle rock materials. After 5 cycles (Fig. 6b), a distinct shift emerges: the AF value is suppressed from the outset, and its fluctuations become more erratic. Meanwhile, the RA value exhibits a pronounced increase in the post-peak region, suggesting that shear cracking becomes increasingly dominant as damage accumulates. At 10 cycles (Fig. 6c), the AF trajectory shows a rapid decline immediately following the linear elastic stage, coupled with an earlier onset of RA escalation. This accelerated transition implies that cyclic wetting-drying has weakened the intergranular bonds, facilitating the early activation of shear mechanisms. Following 20 cycles (Fig. 6d), the AF value exhibits moderate-to-high levels during initial loading but decreases sharply once crack propagation initiates. The RA increase occurs earlier than in previous cases, indicating that shear cracking not only dominates but also initiates at lower stress levels. The most dramatic transformation occurs after 30 cycles (Fig. 6e). Here, the AF value remains persistently elevated throughout the loading process, with only minor oscillations, while the RA value shows minimal variation. This high AF suggests that tensile cracking becomes the prevailing failure mode, a direct consequence of severe microstructural degradation and weakened interparticle cohesion.

**Fig. 7:**
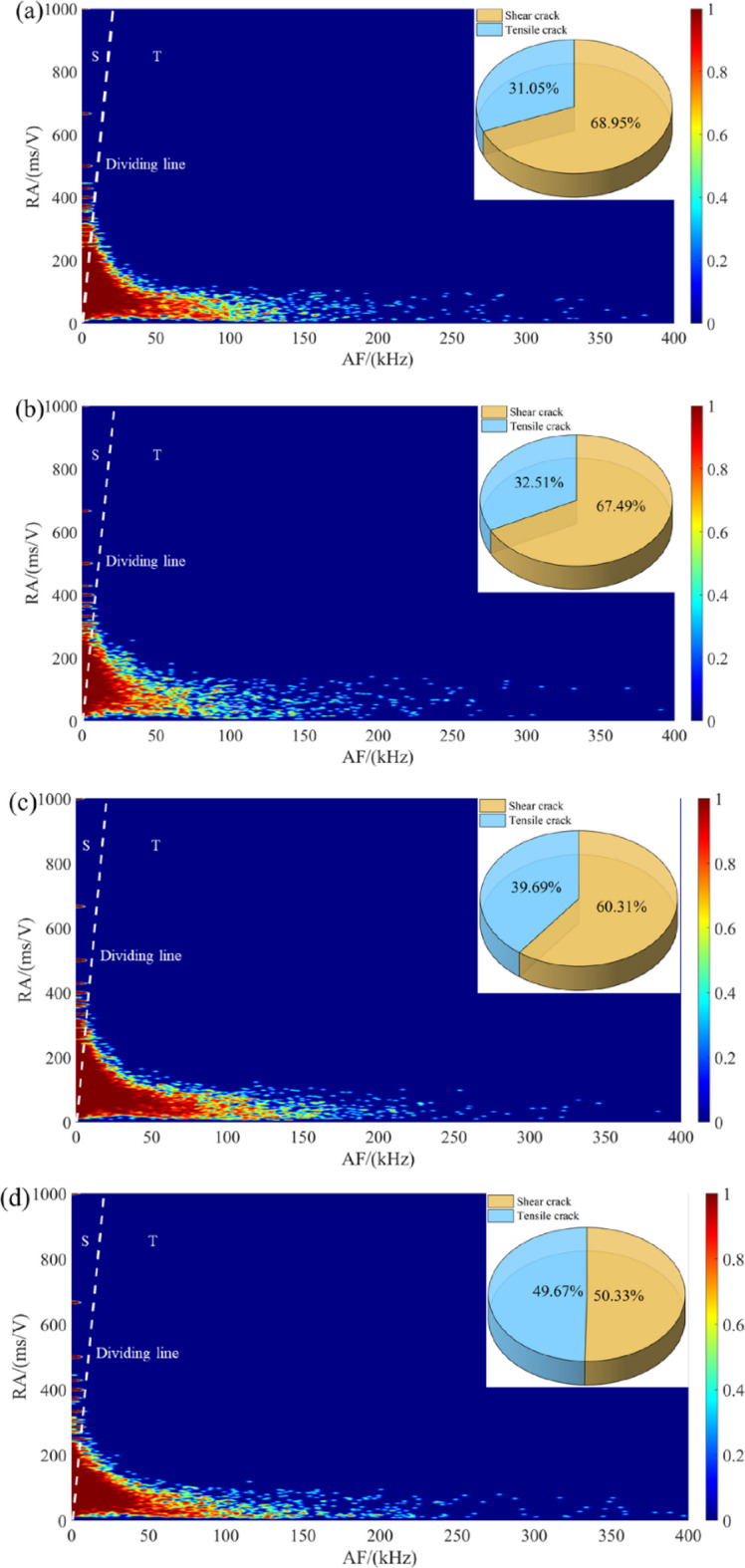

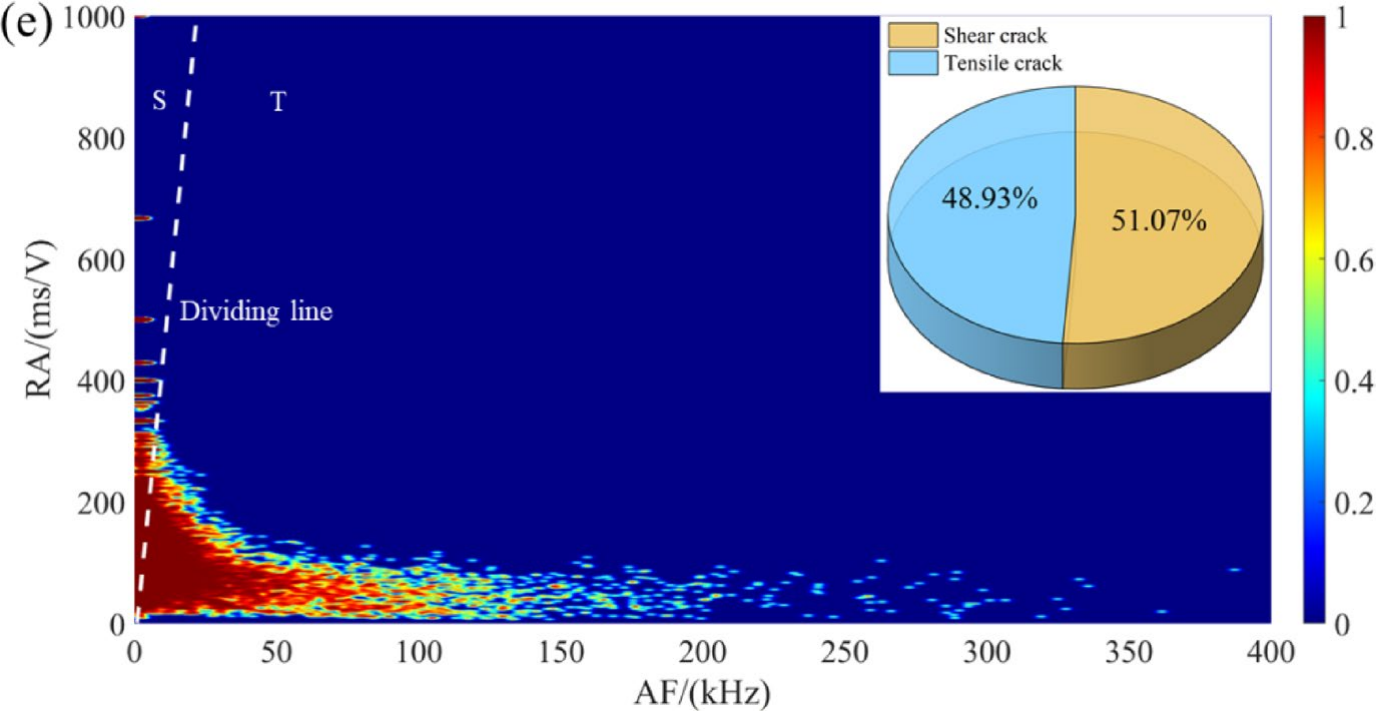
Distribution characteristics of RA-AF for sandstone under different dry-wet cycles: (**a**) *N* = 0, (**b**) *N* = 5, (**c**) *N* = 10, (**d**) *N* = 20, and (**e**) *N* = 30.

To further investigate the failure mechanism of crack rupture, the RA-AF distribution of sandstone under different dry-wet cycles is shown in Fig. 7. In accordance with the previous research, the threshold value of *K* = RA/AF = 70 is employed to differentiate tensile and shear failure modes^23^. It is observed that shear failure is the dominant mode regardless of the number of dry-wet cycles. Nevertheless, the proportion of tensile cracks follows a nonlinear upward trend, increasing from 31.05% to 48.93%. This increase is characterized by an initial rapid ascent, a subsequent deceleration, and a marginal decline between the 20 and 30 cycle intervals. This phenomenon is primarily attributed to the evolving microstructural response to cyclic dry-wet. The initial proliferation of tensile cracks is facilitated by specimen swelling and shrinkage, which induces microcrack nucleation and growth, leading to a transition from a dense to a more porous fabric and a consequent concentration of tensile strain. With continued cycling, the system approaches a quasi-stable state as existing cracks coalesce or close, a process concurrent with internal stress redistribution that mitigates further tensile crack propagation. The eventual slight reduction in tensile crack proportion is attributed to long-term degradation mechanisms, such as surface particle spalling and interfacial bond weakening.

#### Final failure patterns

**Fig. 8:**
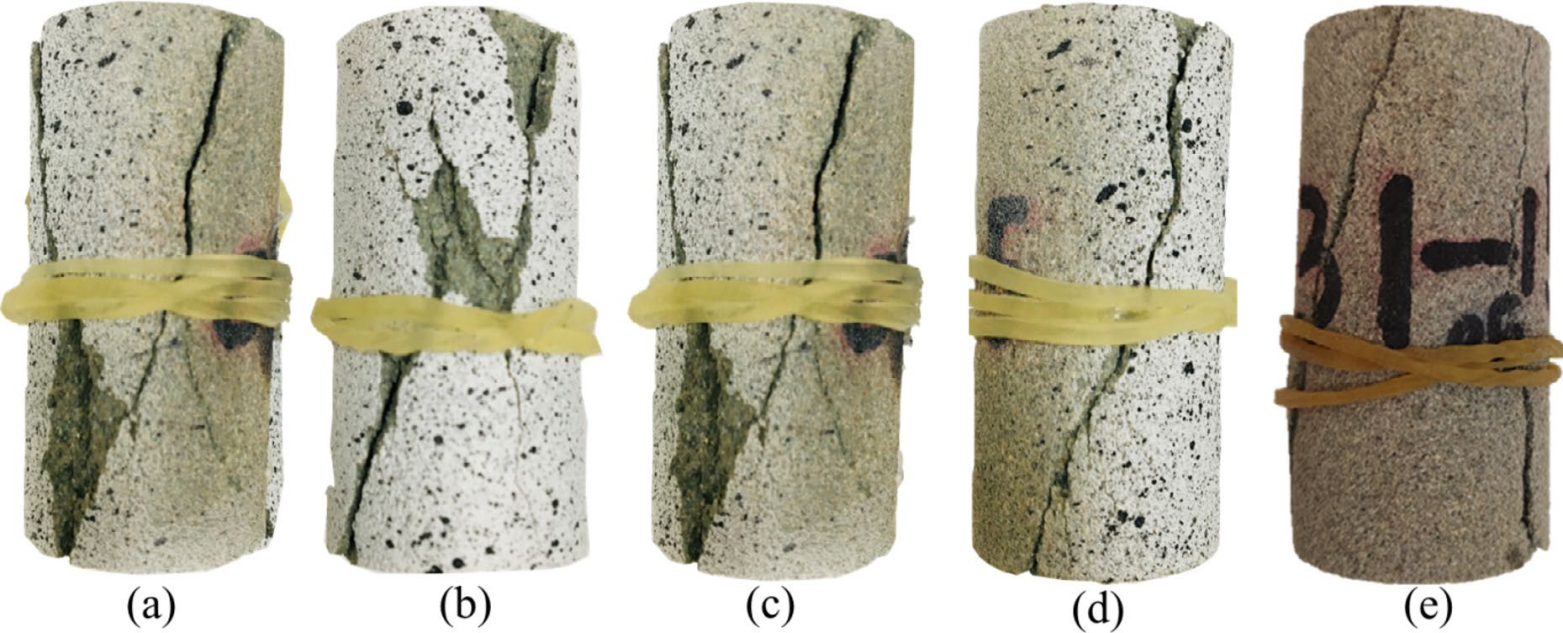
Final failure patterns of sandstone under different dry-wet cycles: (**a**) *N* = 0, (**b**) *N* = 5, (**c**) *N* = 10, (**d**) *N* = 20, and (**e**) *N* = 30.

The typical macroscopic failure patterns of sandstone under different numbers of dry-wet cycles are shown in Fig. 8. When the number of dry-wet cycle is relatively small (Fig. 8a and c), the failure mode predominantly exhibited a bifurcated *Y*-shape pattern, accompanied by local flake spalling and small block detachment on the specimen surface. These features suggest that rock retains good structural integrity. After 20 dry-wet cycles (Fig. 8d), the occurrence of local flaking and small-scale block detachment gradually decreases. This phenomenon indicates the weakening of mineral particles interfaces and growth of microcracks due to alternating wetting and drying. After 30 dry-wet cycles (Fig. 8e), the number of branch cracks increases significantly. The failure mode shifts from control of a single dominant crack to multiple splitting mode and the surface integrity deteriorates further.

### Mesostructural characteristics of sandstone under different dry-wet cycles

#### *T*_2_ distribution

The *T*₂ relaxation time was measured to evaluate the interaction between the hydrogen atom and the surrounding environment^24,25^. This parameter is intrinsically correlated with the geometry of the pore size and shape. Within a porous medium such as rock, the pore space is occupied by water, which contains numerous hydrogen nuclei. NMR testing fundamentally relies on detecting the magnetic response of these nuclei. Consequently, the detected signal intensity is proportional to their number. Through quantitative analysis of these NMR signals, key petrophysical properties, including porosity and pore-size distribution, can be determined:

5 $$\frac{1}{{{T_2}}}=\frac{1}{{{T_{2B}}}}+\frac{1}{{{T_{2D}}}}+{\rho _2}(\frac{S}{V})$$

where *T*_*2*_, *T*_*2B*_, and *T*_*2D*_ are the lateral relaxation time, volume relaxation time and diffusive relaxation time, respectively. *ρ*_*2*_ is the relaxation rate of the rock surface. *S*/*V* is the ratio of pore surface area to fluid volume.

Since volume and diffusion relaxation times are not long or even negligible^26^, Eq. (5) is simplified as:

6 $$\frac{1}{{{T_2}}}={\rho _2}(\frac{S}{V})={F_S}\frac{{{\rho _2}}}{{{r_c}}}$$

where *F*_*S*_ is the geometric shape factor, with two common factors: spherical pore factor, *F*_*S*_=2.0, and capillary pore factor, *F*_*S*_=2.0.

**Fig. 9:**
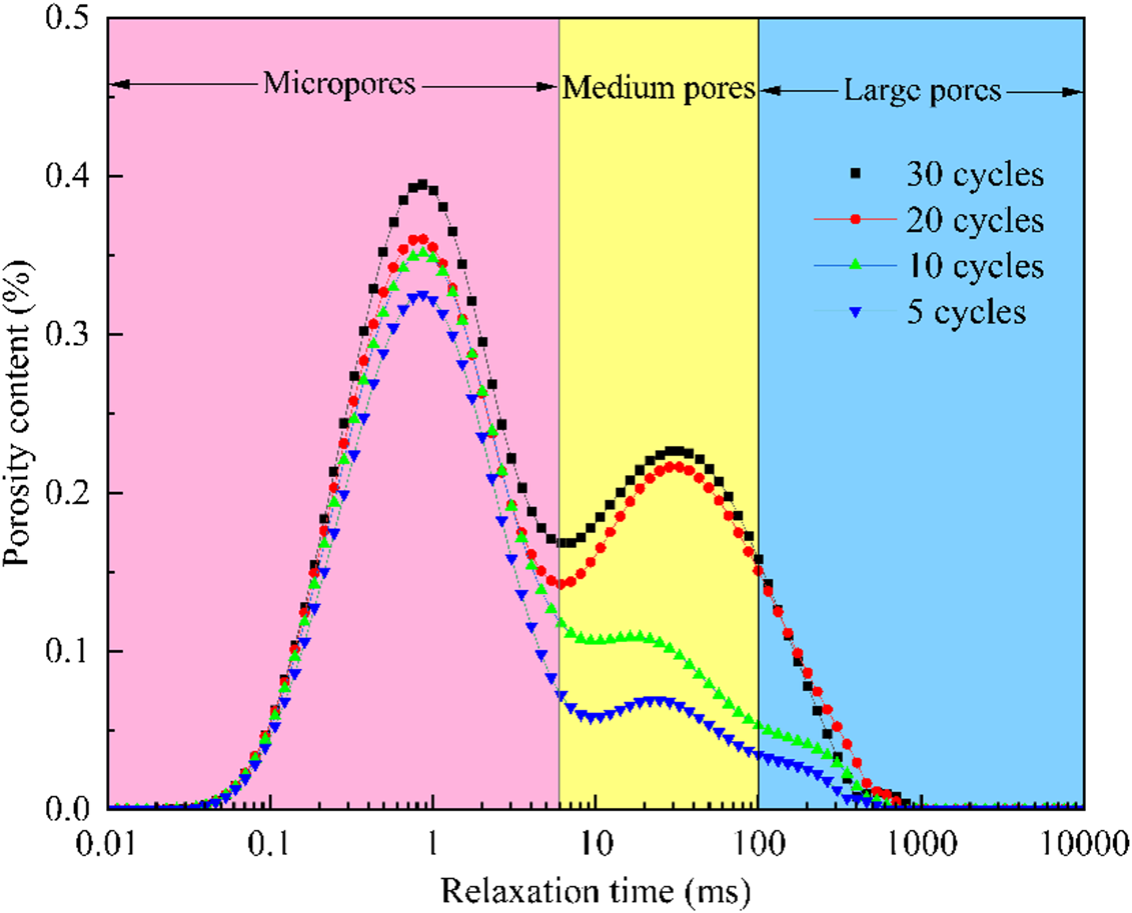
The evolution of *T*_2_ spectrum for sandstone under different dry-wet cycles.

The *T*_2_ spectra of rock samples subjected to varying numbers of dry-wet cycles are depicted in Fig. 9. The spectra consistently exhibit a multi-peak distribution, which is characteristic of a heterogeneous pore structure comprising micropores, mesopores, and macropores. A clear evolution of the *T*_2_ spectra is observed with increasing cycle number. Primarily, the spectral area expands, signifying enhanced pore connectivity. Concurrently, the overall spectral amplitude rises, reflecting an increase in total porosity. A notable transformation is the evolution of the *T*_2_ distribution from unimodal to bimodal, indicating a divergence in the pore-size distribution and an escalation in structural heterogeneity.

To further elucidate the influence of dry-wet cycles on the pore structure, a quantitative analysis of the proportions of micropores, mesopores, and macropores is calculated as listed in Table 1. When the number of dry-wet cycles increases from 5 to 30, the spectrum area of total pores exhibits a remarkable growth from 1170.79 to 5464.24. In detail, the number of dry-wet cycles increases from 5 to 10, 10 to 20, and 20 to 30, and the corresponding growth rates are 65.9%, 119.0%, and 28.4%, respectively. The results indicate that the effect on pore expansion is most pronounced between 10 and 20 cycles. With respect to pore composition, a substantial reduction in the percentage of micropores from 8.09% to 0.24% was observed. Conversely, the proportion of macropores increases from 50.78% to 65.30%. The proportion of mesopores fluctuates between 34% and 41% with a slight decrease, but their spectrum area increases significantly. These findings suggest that the proliferation of mesopores and macropores is the primary contributor to the overall increase in pore area. This phenomenon is attributed to the chemical reaction between H⁺ and carbonate minerals, which undermines the cementation of mineral particles. Concurrently, stress relaxation facilitates the nucleation of micropores. These nascent pores then enable water ingress via capillary action, triggering ion exchange and mineral dissolution. This leads to the progressive erosion and coalescence of micropores into larger voids. This process culminates in the development of a multiscale, interconnected seepage network.

**Table 1:** Percentage of different pores for sandstone under different dry-wet cycles.

Number of dry-wet cycles	Percentage of micropores	Percentage of mesopores	Percentage of large pores	Total pore area
5	8.09%	41.13%	50.78%	1170.79
10	5.77%	38.02%	56.21%	1942.57
20	2.71%	41.11%	56.18%	4255.02
30	0.24%	34.46%	65.30%	5464.24

#### The evolution of porosity and pore-throat distribution

The results from the *T*_2_ spectra and pore-size distribution analyses reveal the progressive evolution of the pore structure of sandstone during different dry-wet cycles. However, *T*_2_ spectrum analysis effectively characterizes the relative changes among pores of different scales, it does not directly quantify the overall increase in the rock’s total pore volume. Porosity, defined as the ratio of pore volume to total volume in a porous medium, is therefore used to measure this absolute change:

7 $$\varphi =\frac{{{V_v}}}{{{V_t}}}$$

where *V*_*v*_ is the pore volume and *V*_*t*_ is the total volume.

**Fig. 10:**
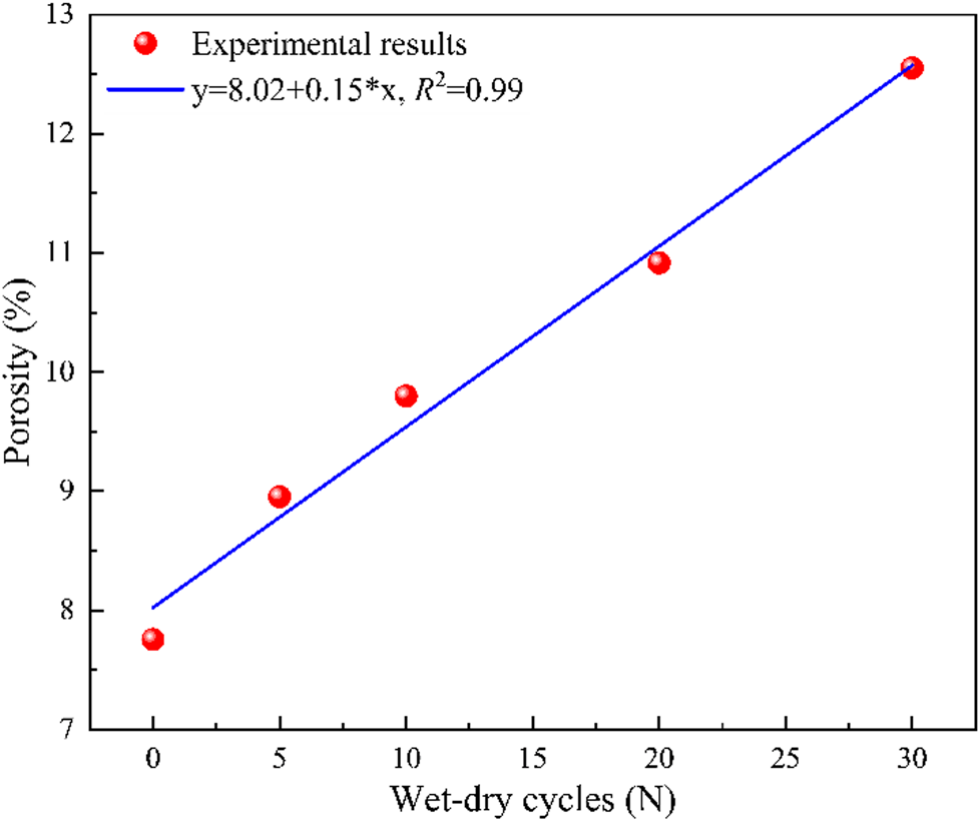
The evolution of porosity for sandstone under different dry-wet cycles.

Figure 10 shows the evolution of porosity for sandstone under varying numbers of dry-wet cycles. The porosity of the sandstone exhibits a general upward trend with increasing cycles. When the number of dry-wet cycles is low, the porosity increases rapidly due to repeated water infiltration and drainage which promote local crack propagation, pore enlargement, and partial pore closure. This stage is characterized by irregular pore geometry and unstable connectivity, leading to pronounced fluctuations in total porosity. When the number of dry-wet cycles increases from 10 to 20, the rate of porosity tends to be slow. When the number of dry-wet cycles exceeds 20 cycles, a proliferation of microcracks occurs, and porosity continues to increase. This is accompanied by a progressive change in pore shape and size, the reopening of sealed pores, and the development of new cracks within the rock matrix. Ultimately, the relationship between porosity and cycle number follows an exponential trend, indicative of a nonlinear pore evolution mechanism driven by the interplay among hydraulic cycling, mineral dissolution, and accumulated microstructural damage.

**Fig. 11:**
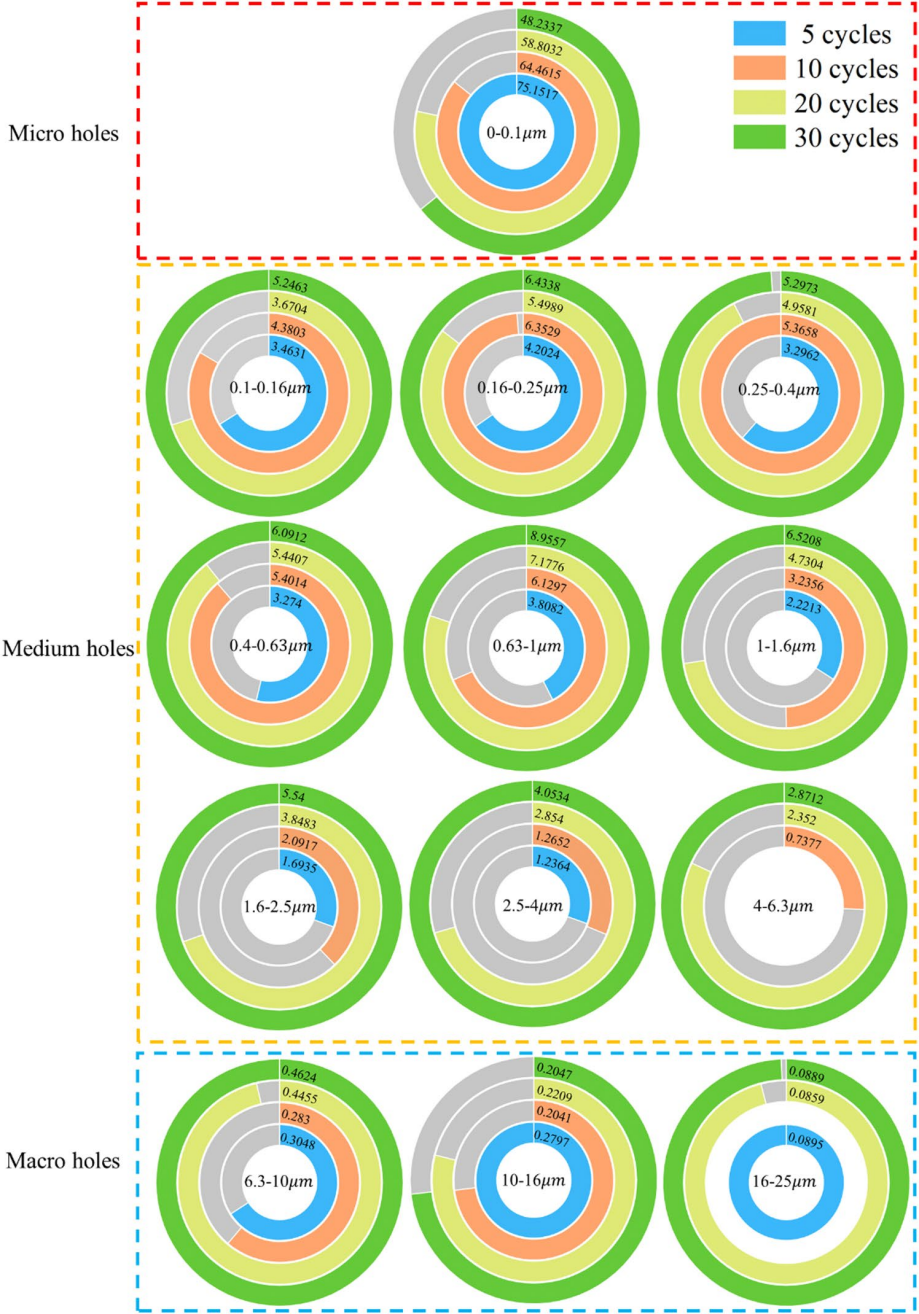
The distribution of various pore throat sizes for sandstone under different dry-wet cycles.

The pore-throat distribution describes the size and arrangement of internal pores and throats within porous media^27^. Figure 11 shows the evolution of pore-throat distribution in typical sandstone under different dry-wet cycles, which can be divided into three stages: the low-cycle condition (*N* = 5 and 10), the middle-cycle condition (*N* = 10 and 20), and the high-cycle condition (*N* = 20 and 30). During the initial stage, pore throats undergo slight deformation due to water evaporation and rewetting, while maintaining a relatively high proportion of micropore throats with regular shapes. As the cycles progress, the proportion of micropore throats decreases and shapes become increasingly irregular. Repeated water evaporation and wetting cause cracks to form at throat edges. After multiple cycles, the existing throats merge or split, forming new cracks.

From a size-based perspective, pore throats are categorized as micropores (0–0.1 μm), mesopores (0.1–6.3 μm), and macropores (6.3–25 μm)^28^. The uneven, irregular, and multi-peak characteristics of the distribution histogram reflect the complexity of the pore-throat structure. As the number of dry-wet cycles increases, the proportion of micropore throats decreases due to water-rock reactions expand some micropores. The proportion of mesopore throats remains relatively stable, suggesting a correlated structure with a homogeneous yet rough surface. The ratio of pore-throats in the range of 0.63–1 μm reaches its maximum at 30 cycles, indicating a more complex structure. The most significant change in the pore-throat ratio occurs during the transition from micropores to mesopores, revealing substantial differences in distribution. This is primarily due to the repetitive water entry and exit during dry-wet cycles, which cause some throats to expand. In contrast, others shrink or close, particularly affecting micro- cracks or weakly connected throats. In contrast, some larger pore-throats close during the cycles, especially those surrounded by more compact material. The proportion of macropore is minimal and discrete, indicating high heterogeneity. The sizes concentrated between 6.3 μm and 1 μm, which further confirms the increasing structural complexity.

#### Fractal characteristics of pore structure

The fractal characteristics of pores and throats are key parameters that characterize the complexity and self-similarity of the pore network across various scales^29–31^. Unlike simple geometry, the pore and throat structures within porous media are highly complex and irregular. Consequently, a higher fractal dimension signifies a more intricate pore structure, and vice versa. In addition, the fractal dimension can indirectly reflect the pore size distribution, geometry, and connectivity. According to fractal theory, the number of pores (*N*) with a radius greater than *R* follows a power-law relationship^32–36^.

8 $$P(r)=N({\mathrm{r}})=a \times {{\mathrm{r}}^{ - D}}$$

where *N*(*> r*) is the number of pores larger than the radius *r*, *P*(*r*) is the pore size distribution density function, *a* is the pore shape coefficient, and *D* is the fractal dimension.

The density function *P*(*r*) of the pore size distribution can be obtained by taking the derivative of both sides of Eq. (8).

9 $$P(r)=\frac{{dN( r)}}{{dr}}= - Da{r^{ - D - 1}}$$

In terms of the cylindrical volume *V*(*R*)*=πr*^*2*^*h*, assuming a constant pore height, the cumulative pore volume less than the radius *R* is given by:

10 $$V(r)=\int_{{{r_{\hbox{min} }}}}^{r} {P(r)a{r^3}} dr$$

where *V* (*< r*) is the cumulative pore volume with a pore diameter less than *r*.

Substituting Eq. (9) into Eq. (10), the integral is obtained:

11 $$V(r)={a_1}({r^{3 - D}} - r_{{\hbox{min} }}^{{3 - D}})$$

where *a*_1_ is the constant of proportionality, ${a_1}=\frac{{{a^2}D}}{{D - 3}}$.

Similarly, the expression for the total volume of the hole is:

12 $${V_t}={a_1}(r_{{\hbox{max} }}^{{3 - D}} - r_{{\hbox{min} }}^{{3 - D}})$$

where *V*_*t*_ is the total volume of the pore.

Therefore, the expression for the volume fraction of pores with a diameter less than *r* is:

13 $${S_V}=\frac{{V(r)}}{{{V_t}}}=\frac{{{r^{3 - D}} - r_{{\hbox{min} }}^{{3 - D}}}}{{r_{{\hbox{max} }}^{{3 - D}} - r_{{\hbox{min} }}^{{3 - D}}}}$$

where *S*_*V*_ is the ratio of the volume of pores with pore diameter less than *r* to the total volume.

Since *r*_*max*_ is much larger than *r*_*min*_, Eq. (13) can be simplified as:

14 $$W=\frac{{{r^{3 - D}}}}{{r_{{\hbox{max} }}^{{3 - D}}}}={(\frac{r}{{{r_{\hbox{max} }}}})^{3 - D}}$$

Taking the logarithm of both sides of Eq. (14) at the same time to obtain the fractal dimension expression of the pore structure.

15 $$Lg(W)=(D - 3)lg({r_{\hbox{max} }})+(3 - D)lg(r)$$

**Fig. 12:**
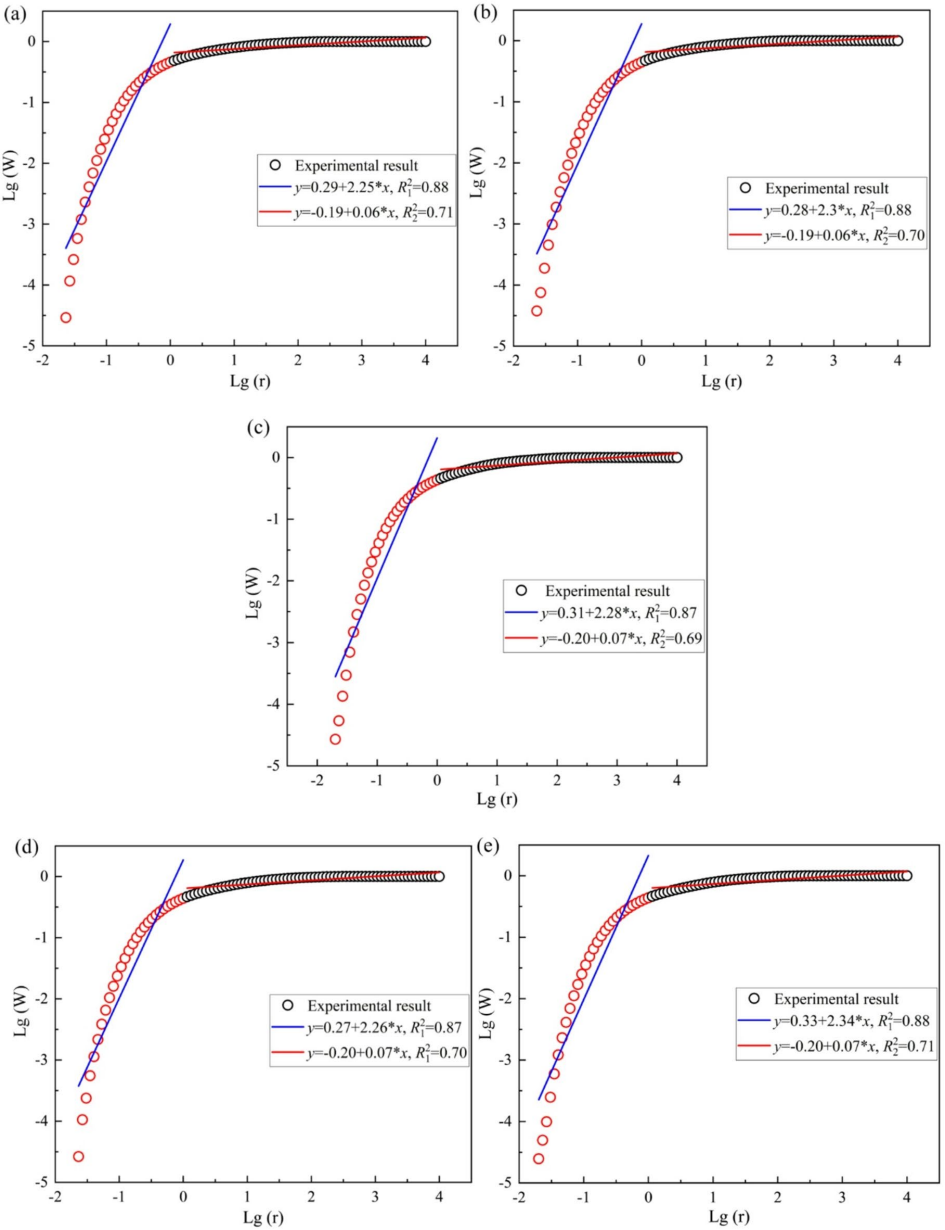
The fractal characteristics of pore size for sandstone under different dry-wet cycles: (**a**) *N* = 0, (**b**) *N* = 5, (**c**) *N* = 10, (**d**) *N* = 20, and (**e**) *N* = 30.

The porosity of the sandstone was determined from the total area of the *T*_2_ spectrum, as the integral area is directly proportional to the pore volume. The fractal characteristics of the pore structure under varying dry-wet cycles were obtained by fitting the data to Eq. (15). Figure 12 shows the fitting curves for specimens subjected to 0, 5, 10, 20, and 30 dry‑wet cycles. An inflection point is consistently observed at Lg(*T*₂) ≈ 0 across all cases, distinguishing the pore regimes into micro‑mesopores (*T*₂ < 1 ms) and macropores (*T*₂ > 1 ms). The fractal dimensions for these two regimes (*D*₁ for micro‑mesopores and *D*₂ for macropores) were calculated from the slopes of the fitted lines.

For the untreated specimen (Fig. 12a), the fitting yields a macropore fractal dimension *D*₂ = 2.25, indicating a rough and complex pore surface in the macropore region. Conversely, the micro‑mesopore region exhibits a fractal dimension *D*₁ = 0.06. As the value falls outside the theoretical range of 2–3, suggesting that a single fractal model is inadequate for characterizing pores smaller than 6.3 μm. After 5 cycles (Fig. 12b), the fractal dimension increases slightly to *D*₂ = 2.30, reflecting enhanced surface roughness and structural complexity. The micro‑mesopore region maintains a low D₁ value of 0.06, confirming that fractal scaling does not apply to this pore size range. At 10 cycles (Fig. 12c), *D*₂ decreases marginally to 2.28, while *D*₁ remains at 0.07. The slight reduction in *D*₂ may indicate the onset of pore coalescence, which tends to smooth the pore surface as smaller pores merge into larger ones. Following 20 cycles (Fig. 12d), a more pronounced decrease in *D*₂ is observed, reaching 2.31. Although this value remains within the typical range for porous media, the variation suggests progressive simplification of the pore surface as connectivity improves. The most significant change occurs after 30 cycles (Fig. 12e), where *D*₂ attains its minimum value of 2.34. Interestingly, this represents a slight increase compared to Fig. 12d, but overall, the *D*₂ values across Fig. 12a and e range from 2.25 to 2.34, all indicating highly rough pore networks. The micro‑mesopore region consistently yields *D*₁ values between 0.06 and 0.07, confirming that the fractal model is only applicable to the macropore regime. As the number of dry-wet cycles increases, the *D*_2_ value exhibits a downward trend. This trend suggests that the dry-wet cycles enhance pore connectivity, thereby accelerating the deterioration of the sandstone’s pore network structure.

### Microstructural characteristics of sandstone under different dry-wet cycles

**Fig. 13:**
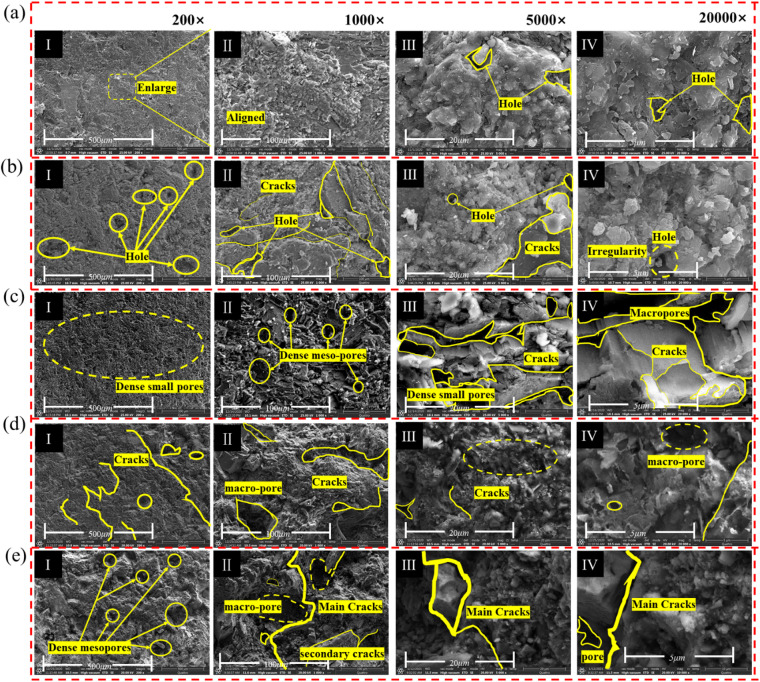
SEM observation of sandstone under different dry-wet cycles: (**a**) *N* = 0, (**b**) *N* = 5, (**c**) *N* = 10, (**d**) *N* = 20, and (**e**) *N* = 30.

To elucidate the evolution of pore morphology, fracture development, and mineral interface damage in sandstone under varying dry-wet cycles, SEM was performed at multiple magnifications, as shown in Fig. 13. In its initial state, the microstructure is characterized by a dense skeletal structure of tightly bound particles, with minimal fracture spacing and only sporadic micropores and microcracks observed on the particle surfaces (Fig. 13a).

After five dry-wet cycles, microstructural degradation becomes evident (Fig. 13b). Small dissolution pits emerge and proliferate, accompanied by the extension and interconnection of microcracks. This process involves the erosion and dissolution of cementing materials and fine particles, resulting in enlarged pores and the formation of new cracks. Following ten cycles, the degradation intensifies (Fig. 13c), with more pronounced dissolution and cracking on particle surfaces and the coalescence of pores and cracks into larger fractures, indicating an acceleration of the damage. Furthermore, the formation of surface debris, coupled with an increased prevalence of pores and cracks, significantly compromises the inter-particle bonding strength. At 2000× magnification (Fig. 13d-III), more significant dissolution and cracking are evident on the particle surfaces.

After 20 cycles, hydration-induced volumetric expansion of minerals and the matrix generates uneven deformation due to disparate expansion coefficients, precipitating the formation of significant dissolution pores (Fig. 13d). At 2000× magnification, the initiation of new secondary cracks and increasingly irregular pore morphologies are discernible. The degradation culminates after 30 cycles in a severely compromised microstructure (Fig. 13e). The lubricating effect of interlayer-free water reduces interparticle friction, further promoting pore growth and connectivity. The expansion of cracks and macropores renders the sandstone structure highly porous and loose. High-magnification imaging reveals an extensive pore network formed by the interconnection of large pores with main fractures, while surface examinations show pronounced cracking, debris, and indistinct mineral grain edges, signifying advanced dissolution and structural weakening.

To quantitatively analyze the pore evolution of sandstone under different dry-wet cycles, the ImageJ software version 1.53t (https://imagej.net/ij/) was employed for image preprocessing and pore analysis. The SEM images were first converted to grayscale and denoised. Subsequently, pore regions were extracted as binary images via threshold segmentation, clearly distinguishing the pores from the matrix. These regions were further refined using morphological operations to obtain connected domains. Finally, the Feret diameter was calculated for each connected domain, followed by statistical analysis.

The Feret diameter is the maximum outer diameter of a pore, and it is usually defined by measuring the maximum width of the pore in different directions. The calculation formula is:

16 $${D_{F,i}}=\mathop {\hbox{max} }\limits_{\theta } \left( {\mathop {\hbox{max} }\limits_{{p \in {R_i}}} p \cdot {{\mathbf{u}}_\theta } - \mathop {\hbox{min} }\limits_{{p \in {R_i}}} p \cdot {{\mathbf{u}}_\theta }} \right) \cdot s$$

where, ${D_{F,i}}$ represents the Feret diameter of the *i*-th pore, ${u_\theta }$ is the unit vector of the direction angle $\theta$, and *p* is the pixel point of the pore boundary.

**Fig. 14:**
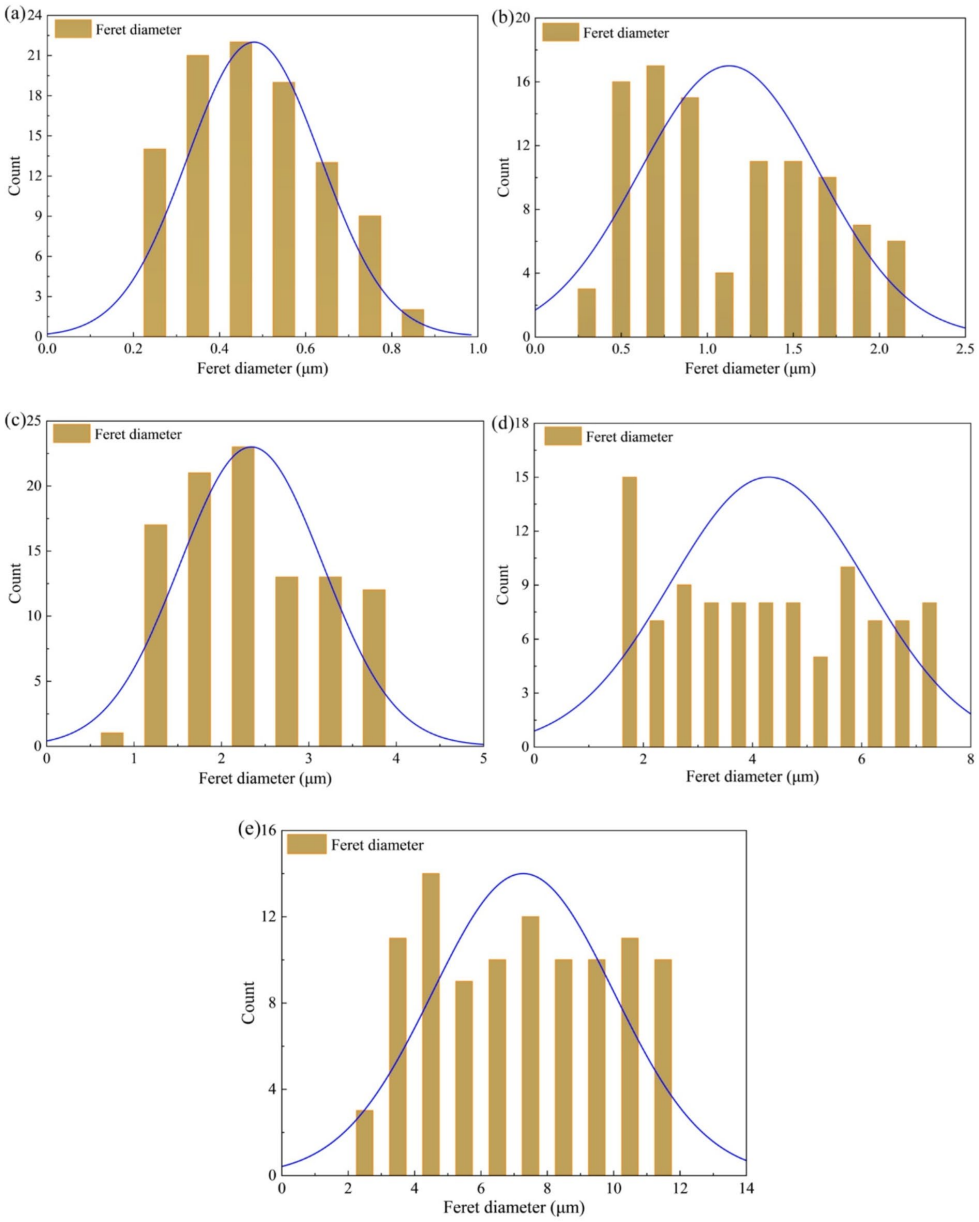
Feret diameter of sandstone under different dry-wet cycles: (**a**) *N* = 0, (**b**) *N* = 5, (**c**) *N* = 10, (**d**) *N* = 20, and (**e**) *N* = 30.

Figure 14 shows the Feret diameter distribution of pores in sandstone subjected to different dry-wet cycles. For the sample subjected to 5 cycles (Fig. 14a), the Feret diameters are primarily distributed between 0.21 μm and 1.27 μm, with a mean of approximately 0.62 μm, confirming a micropore-dominated structure. After 10 cycles (Fig. 14b), the diameter range expands to 0.28–5.23 μm, and two distinct populations (centered around 0.5 μm and 2.5 μm) are observable, indicating the coexistence of micropores and mesopores. After 20 cycles (Fig. 14c), the distribution becomes broader and shifts towards larger sizes, with a mean diameter of 4.8 μm. In the samples subjected to 20–30 cycles (Fig. 14d and e), the Feret diameter extends to 10.20 μm and 14.36 μm, respectively, with mean values reaching 6.5 μm and 8.2 μm, signifying a transition to a macroporous network. This rightward shift in the pore size distribution corroborates the pore enlargement trend observed in the area histograms (Fig. 14) and is consistent with the *T*₂ spectra evolution measured by NMR (Fig. 8).

## Discussions

### Precursor identification of the variance of different AE parameters

**Fig. 15:**
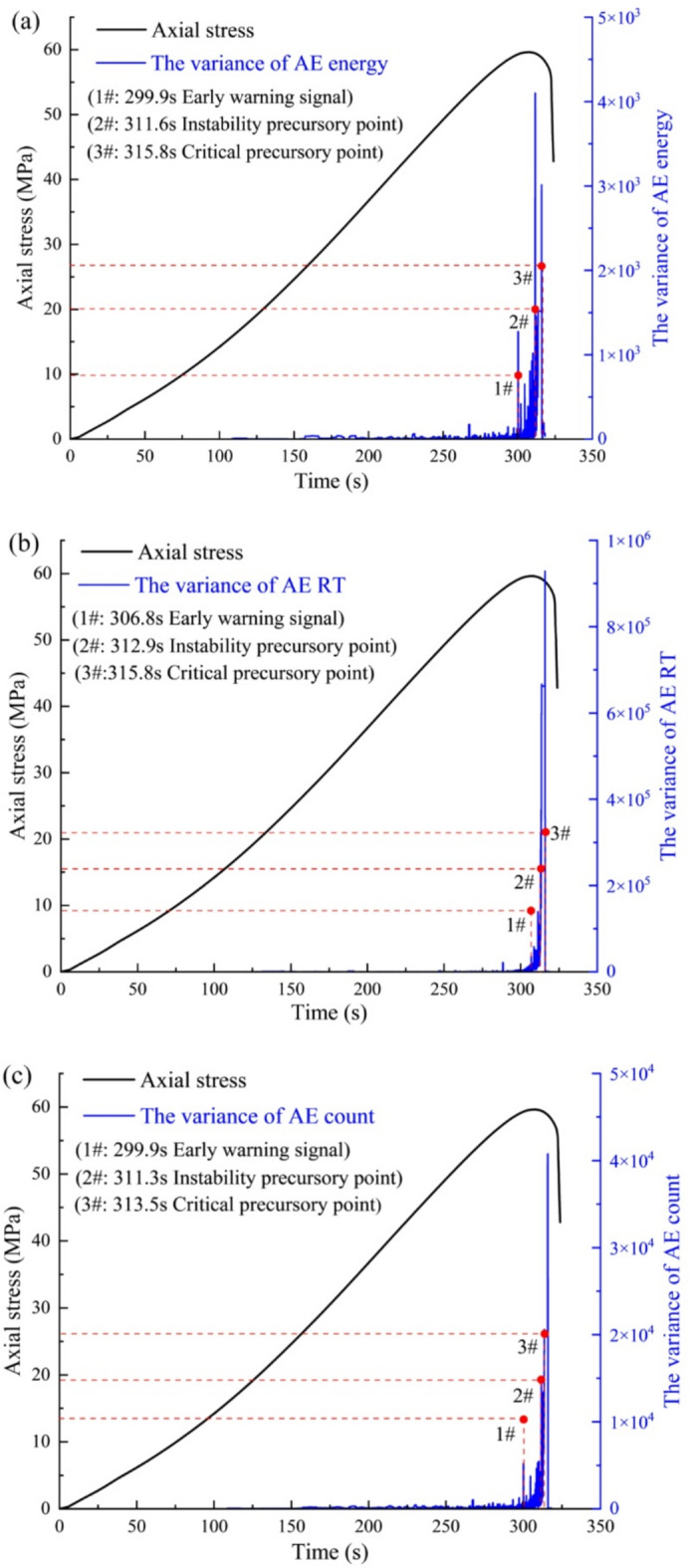
The evolution of different AE parameters variance for typical sandstone: (**a**) AE energy, (**b**) AE RT, and (**c**) AE count.

Figure 15 illustrates the variance curves of AE parameters for a typical dry-wet condition (*N* = 20). A general consistency in the variance trends across the different AE parameters is observed. On a more detailed level, the fluctuation amplitude of the RT surpasses that of both the AE count and AE energy. Figure 15a reveals a distinct critical slowing down phenomenon, coinciding with a sharp increase in variance amplitude. We define the early-warning point as the instance characterized by a sudden surge in variance amplitude coupled with a deceleration in the recovery rate from perturbations. After this initial surge, the instability precursor point is identified by a secondary increase in variance amplitude, also accompanied by a slowing perturbation rate. With continued loading, the variance amplitude escalates, and the perturbation rate diminishes significantly during the propagation and coalescence of macro-cracks, marking the critical point of final failure. A comparative analysis of the precursor identification shows that the variances of AE count and AE energy provides an early-warning 6.9 s earlier than the RT variance. Similarly, the instability precursor indicated by the AE count precedes those of AE energy and RT by 0.3 s and 1.6 s. Unlike the varying lead times for the early-warning and instability precursors, the precursor time to the final failure point is nearly identical across all three AE parameters.

### Cracking characteristics at different loading stages based on RA-AF

**Fig. 16:**
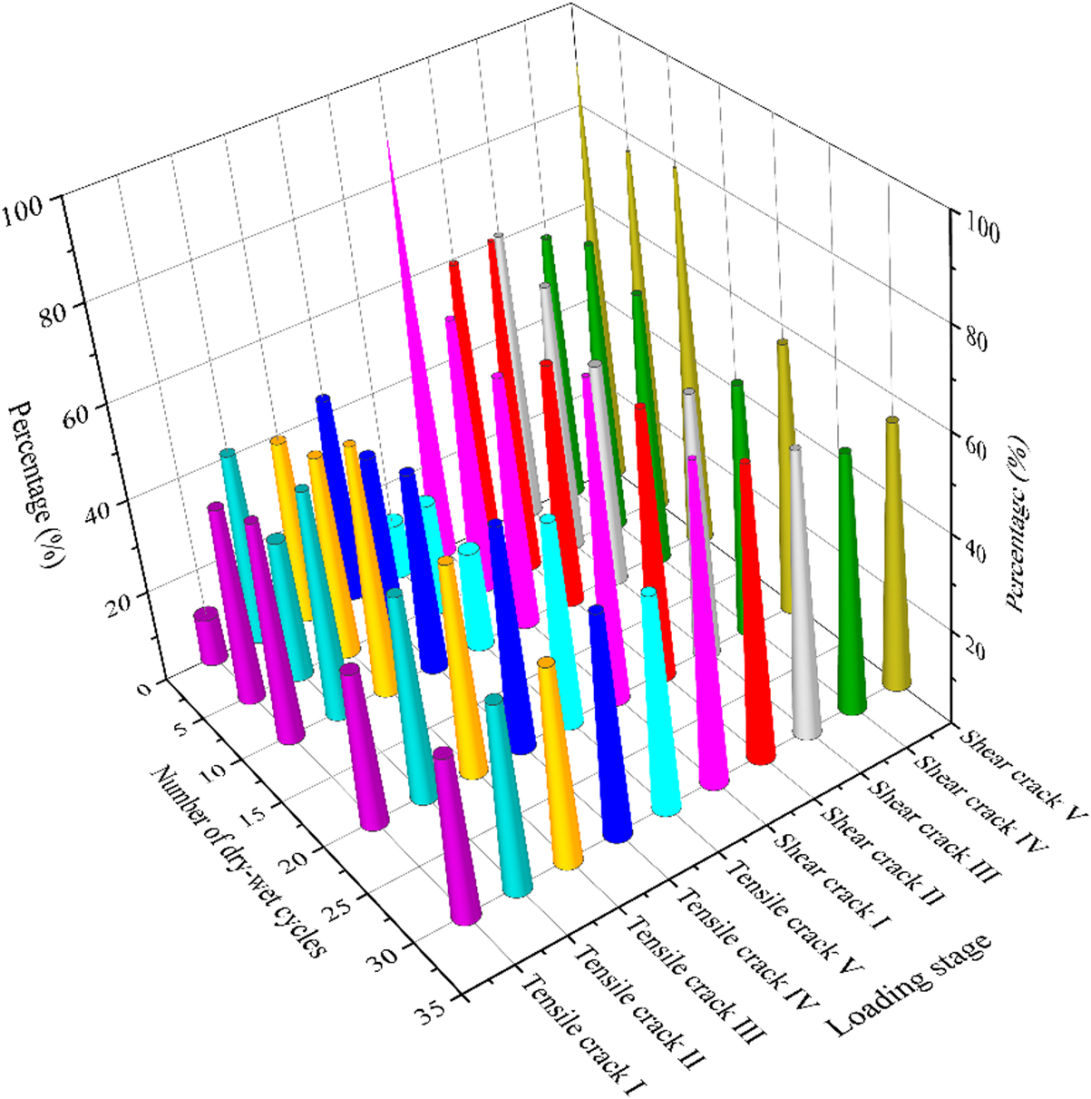
The evolution of microcracks for sandstone under different loading stages.

The variations in the ratios of tensile and shear microcracks in samples at each failure stage under different dry-wet cycling conditions are plotted in Fig. 16. From Fig. 16, it can be observed that with the increase in the number of dry-wet cycles, the proportion of tensile cracks changes from a rapid to a slow growth trend, while the proportion of shear cracks decreases accordingly. In the early stages of wet-dry cycling, water repeatedly enters and exits the pore and microcrack structures of the rock mass, leading to swelling and shrinkage effects in mineral particles. This weakens the bonding forces among mineral particles, causing previously closed or semi-closed microcracks to gradually open and extend along the original structural weak planes. As a result, crack propagation primarily manifests as the rapid initiation and development of tensile cracks, with a relatively fast growth rate. From 0 cycle to 5 cycles, the proportion of tensile cracks increases significantly in most stages. As the number of dry-wet cycles further increases to 10 and 20 cycles, the growth trend of tensile cracks gradually slows down, exhibiting certain fluctuations. After 10 cycles, the proportion of tensile cracks in Stage II of sample increases to 48.41%, but by 20 cycles, it drops back to 43.16%. The proportion of tensile cracks in Stage III is 52.88% after 10 cycles and decreases to 44.69% after 20 cycles. After 30 wet-dry cycles, the repeated opening, closing, and re-expansion of microcracks. The internal structural integrity of the rock mass significantly decreases, and the connectivity between cracks increases. This results in a more stable crack ratio distribution in each stage, with the proportions of tensile and shear cracks becoming more balanced. The proportion of shear cracks ranges from 53.44% to 65.90%, while the proportion of tensile cracks ranges from 34.10% to 45.01%, both showing a more balanced distribution.

**Fig. 17:**
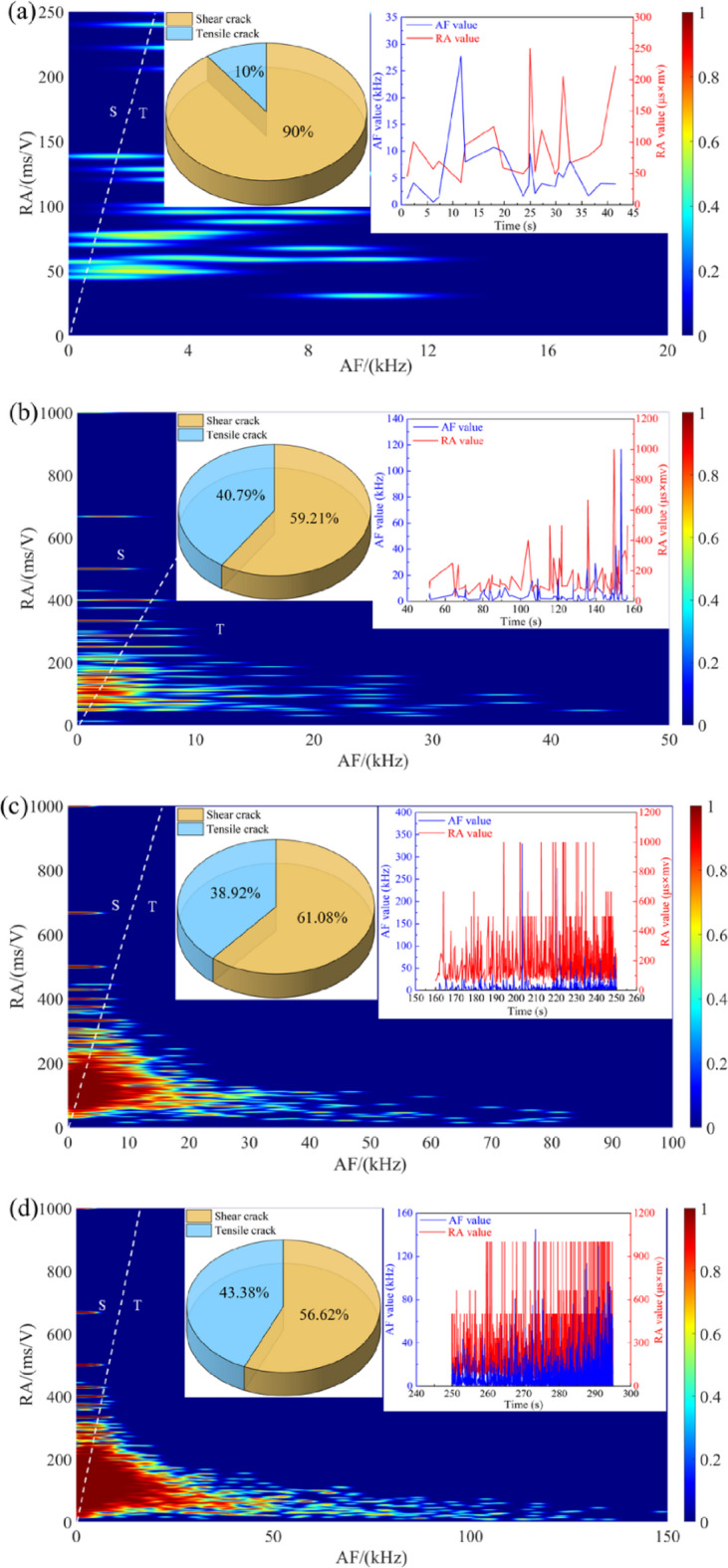

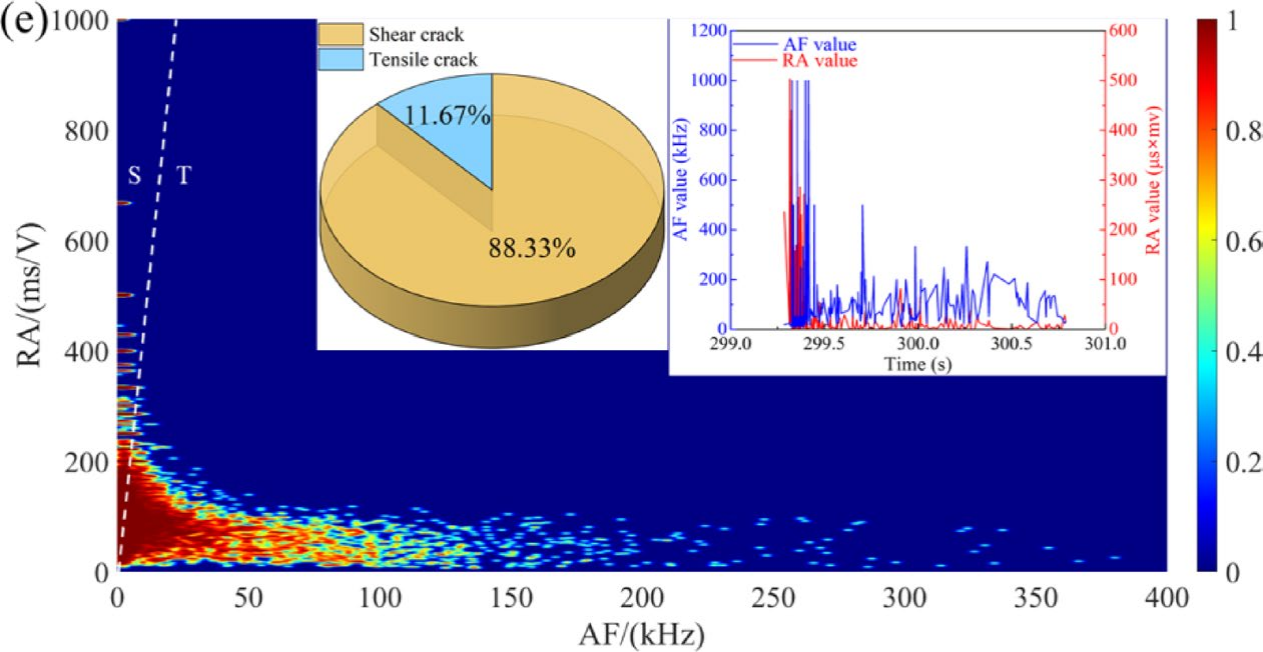
The evolution of microcracks for the typical sandstone under different stages: (**a**) compaction stage, (**b**) linear elastic stage, (**c**) stable crack propagation, (**d**) unstable crack propagation, (**e**) post-peak stage.

In order to conduct a detailed analysis of the evolution characteristics of microcracks in sandstone under different loading stages, Fig. 17 shows the evolution of microcracks modes by statistically presenting the proportions of shear and tensile microcracks. In the specimen subjected to 5 cycles, a pronounced increase in tensile cracks (41.67%), is observed in the compaction stage, implying that tensile microcracks form internally, initiating tensile failure early in the loading process. Subsequently, the failure mode transitions to shear-dominated, with the proportion of shear cracks increasing to 70% in Stage II and 76.26% in the post-peak stage (Stage V). For the specimen subjected to 10 cycles, the distribution of tensile and shear cracks achieves a more equitable balance. Notably, the tensile crack proportion remains within a narrow 45%−53% range from Stage II to Stage IV, suggesting that multiple cycles amplify the role of tensile failure by reducing inter-particle bonding. Nevertheless, the ultimate failure is governed by shear slip, as evidenced by the shear crack proportion rising to 78.78% in the final stage. With further increase of cycles, it is observed that the proportion of the shear crack is generally decreased. This trend is most pronounced in Stages III and V, where the tensile crack proportion consistently exceeds 40%, indicating that progressive dry-wet cycles degrade the specimen’s fabric, thus making tensile failure increasingly prominent. This indicates that the shear crack remains the primary failure mechanism, its dominance is progressively attenuated with increasing cycles.

### Damage mechanism of sandstone subjected to dry-wet cycles

**Fig. 18:**
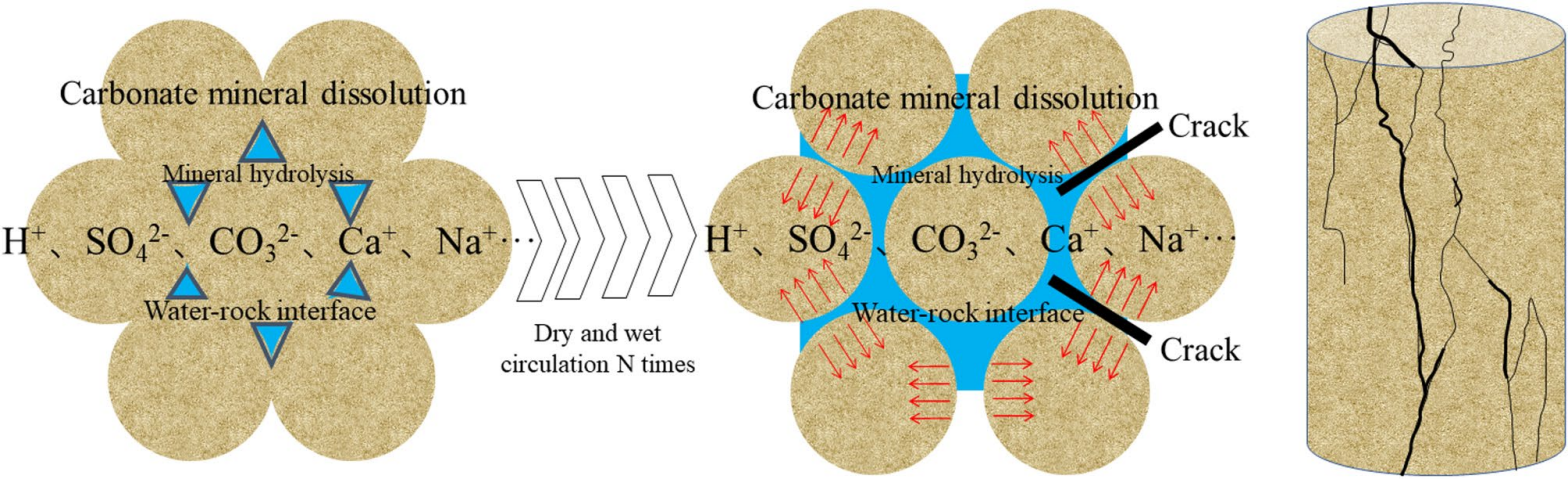
Deterioration mechanism of sandstone subjected to dry-wet cycles.

It is well known that rocks are composed of various minerals, and their mechanical properties are mainly influenced by the mineral composition and the adhesion between the particles. The water environment has both physical and chemical effects on rocks. Physically, water acts as a lubricant at the interface between the particles, reducing the binding force between the mineral particles and decreasing the friction. Chemically, the interaction between water and rocks leads to changes in the mineral composition of the rocks, thereby affecting their mechanical properties^37,38^. Figure 18 shows the deterioration mechanism of sandstone subjected to dry-wet cycles. At the low-cycle conditions (*N* = 0 and 5), the sandstone exhibits high strength and brittleness. The *T*_2_ spectrum displays a single peak with a high proportion of micropores (Table 1), suggesting a simple pore structure. SEM micrographs (Fig. 13a) corroborate this, revealing tightly bonded particles with minimal cracking. This phenomenon is further confirmed by RA-AF analysis (Fig. 7), which indicates the dominance of shear cracks (approaching 70%). This phenomenon is attributed to the significant and heterogeneous strength difference between mineral particles and their cementing matrix. Consequently, crack propagation necessitates overcoming a high energy barrier, typically resulting in mixed-mode. This mechanism accounts for the progressive crack propagation, which aligns with the significant increase in AE energy observed in Fig. 4.

When the number of dry-wet cycles increases to 10 and 20, a notable transition in the deterioration mechanism is observed. NMR analysis indicates a substantial increase in the proportion of macropores, with the *T*_2_ spectrum evolving from a unimodal to a multimodal distribution (Fig. 9). Concurrently, SEM micrographs display an increased prevalence of intergranular cracks, while RA-AF analysis reveals a significant rise in the proportion of tensile cracks. This evolution signifies that the “hydrolysis-dissolution effect” of water becomes the dominant degradation mechanism^39,40^. Water preferentially attacks and weakens the intergranular cements (e.g., clay minerals, carbonates) relative to the mineral particles themselves (e.g., quartz, feldspar), substantially reducing the fracture toughness of the intergranular bonds. As a result, tensile cracks preferentially propagate along these water-weakened interfaces, and shear crack formation is frequently accompanied by greater local stress concentration^41^.

When the number of cycles is 30, the structural integrity of the sandstone is severely degraded. SEM micrographs reveal indistinct mineral particle edges and a highly disintegrated structure, findings corroborated by NMR data that confirm the dominance of macropores (65.30%). By this stage, the overall strength of the sandstone has deteriorated to such an extent that the energy threshold required for the autonomous propagation of tensile cracks is not achieved during the shear slip. This further explains why the failure mode can remain shear-dominated even under these high-cycle conditions where intergranular bonds are significantly weakened.

## Conclusions

(1) In accordance with the theory of critical slowing down, the pronounced escalation in the variance of AE energy, counts, and rise time prior to rock instability constitutes a robust precursor to failure. Quantitatively, the early warning point is identified between 91.76% and 98.53% of the peak stress, whereas the critical fracture point is consistently observed at stress levels exceeding 99%.

(2) According to RA-AF analysis, the tensile microcracks become increasingly pronounced as the number of dry-wet cycles increases. Quantitatively, the proportion of tensile cracks increases substantially from 31.05% to 48.93%, signifying a transition in the failure mode from shear-dominance to a tensile-shear mixed one. Nevertheless, staged analysis demonstrates that shear mechanisms remain the primary governing factor, even under conditions of high cyclic conditions.

(3) Results from NMR tests demonstrate that the total porosity of the sandstone increases exponentially with the number of dry-wet cycles. The *T*_2_ spectrum evolves from unimodal to bimodal, thereby enhancing the structural heterogeneity of the pore network. This observation is corroborated by fractal analysis, which shows decrease in fractal dimension (*D*_2_) in the macropore region, ranging from 2.25 to 2.34. This decline signifies an enhancement in pore connectivity and an acceleration of the overall structural degradation.

(4) SEM observations the microstructural damage process induced by dry-wet cycles. Under a low number of cycles, the damage is characterized by the formation of dissolution pits and isolated microcracks on mineral particle surfaces. As the number of cycles progresses, inter-particle cementation is substantially compromised, leading to the generation of abundant debris. The edges of mineral grains become indistinct, and microcracks propagate and coalesce into macroscopic fractures.

## Data Availability

The data that support the findings of this study are available from the corresponding author upon reasonable request.

## References

[1] Zhang, G. D., Ling, S. X., Xiao, C. J., Liao, Z. X. & Wu, X. Y. Crack evolution of soft red-bed rock under drying-wetting cycles. *J. Rock Mech. Geotech. Eng.* **17**, 5768–5780 (2025).

[2] Li, H., Zhong, Z. L., Liu, X. R., Sheng, Y. & Yang, D. M. Micro-damage evolution and macro-mechanical property degradation of limestone due to chemical effects. *Int. J. Rock Mech. Min. Sci.* **110**, 257–265 (2018).

[3] Xie, K. N., Jiang, D. Y., Sun, Z. G., Song, Z. Q. & Jiang, X. NMR-based analysis of the effect of moisture migration on sandstone pore structure under alternating wetting and drying conditions. *Rock Soil Mech.* **40**(2), 653–659 (2019).

[4] Song, Y. J. et al. Study on damage characteristics of weak cementation sandstone under drying-wetting cycles based on nuclear magnetic resonance technique. *Chin. J. Rock Mech. Eng.* **38**(4), 825–831 (2019).

[5] Wang, H. S., Bi, J., Zhao, Y., Wang, C. L. & Ma, J. B. NMR-based analysis of the effect of moisture migration on sandstone pore structure under alternating wetting and drying conditions. *Int. J. Min. Sci. Tech.* **34**(8), 1135–1150 (2024).

[6] Zeng, Z. et al. Characterizing imbibition and void structure evolution in damaged rock salt under humidity cycling by low-field NMR. *Eng. Geol.* **328**, 107371 (2024b).

[7] Meng, X. Z. et al. Macro-meso physical and mechanical deterioration properties and damage prediction model of rock under freeze-thaw cycles. *B Eng Geol Environ.* **83**, 452 (2024).

[8] Zhou, Z. L. et al. Dynamic tensile properties of sandstone subjected to wetting and drying cycles. *Constr. Build. Mater.* **182**(10), 215–232 (2018).

[9] Yang, J. Q. et al. Experiment of the dissolution mechanism of basalt under the action of water-rock dry-wet cycles. *Chinese J Rock Mech Eng* **44**(8), 2055–2070 (2025).

[10] Yu, X. W. et al. Cracking of silty mudstone subjected to wetting-drying cycles. *J. Rock Mech. Geotech. Eng.* **17**(7), 4195–4210 (2025b).

[11] Kou, M. M., Liu, X. R., Tang, S. D. & Wang, Y. T. 3-D x-ray computed tomography on failure characteristics of rock-like materials under coupled hydro-mechanical loading. *Theor Appl Fract Mech* **104**, 102396 (2019).

[12] Zhao, Y. B. et al. Effect of dry-wet cycles on the mechanical performances and microstructure of pisha sandstone. *Molecular Structure of Minerals* **28**(06), 2533–2533 (2023). 10.3390/molecules28062533 PMC10055890 36985505

[13] Huang, K. et al. Mechanical behavior and fracture mechanism of red-bed mudstone under varied dry-wet cycling and prefabricated fracture planes with different loading angles. *Theor Appl Fract Mech* **127**, 104094 (2023).

[14] Liu, Y. X., Cheng, J. X., Xu, C. H., Wang, G. & Xu, J. Mechanical behaviors and instability of rocks subjected to hydraulic progressive wetting: Acoustic emission and uniaxial compression experiments. *J. Rock. Mech. Geotech. Eng.* **4**. (2025).

[15] Long, N. Z. et al. Effect of dynamic disturbances and dry-wet cycles on the degradation law and acoustic emission evolution characteristics of argillaceous sandstone: An experimental study. *J. Appl. Geophys.* **241**, 105860 (2025).

[16] Tan, H., Jiang, C., Li, J. T., Wang, M. X. & Shi, Z. M. Acoustic emission response characteristics and fracture behavior of dry-wet red sandstone under multistage fatigue loading. *Theor. Appl. Fract. Mech.* **142**, 105358 (2026).

[17] Lei, R. D., Gu, Q. H., Hu, C., He, P. & Zhou, L. S. Study on acoustic emission signal characteristics and failure precursory recognition of fractured sandstone. *Rock Soil Mech* **46**(7), 2023–2038 (2025).

[18] Yu, H. C. et al. Experimental study on the influence of rock bridge dip angle on creep AE characteristics of double-fractured sandstone. *Eng. Fract. Mech.* **322**(12), 111171 (2025a).

[19] Zeng, Z. et al. Acoustic emission insights into rock salt damage under humidity cycling. *Geoenergy Sci. Eng.* **251**, 213898 (2025).

[20] Liang, X. D. et al. Failure prediction of fissured rock under freeze-thaw cycles based on critical slowing down theory of AE multi-parameter. *Results Eng.* **25**, 103874 (2025).

[21] Shi, Z. M. et al. Laboratory study on AE signals and damage mechanism of rock with thermal storage potential under fatigue loading. *J. Energy Storage* **103**, 114414 (2024).

[22] Yang, H. Z. et al. Predicting the failure of rock using critical slowing down theory on acoustic emission characteristics. *Eng. Fail. Anal.* **163**, 108474 (2024).

[23] Zhou, Z. L., Zhou, T. H., Ullah, B. & Fan, J. L. Investigating crack evolution, and failure precursor warning in sandstones with different water contents from the perspective of tensile-shear crack separation. *Eng. Fail. Anal.* **167**, 108997 (2025).

[24] Deng, X. F. et al. Study on the brittle failure of flawed concrete-sandstone based on entropy theory and AE crack classification. *Theor. Appl. Fract. Mech.* **136**, 104773 (2025).

[25] He, S., Li, M., Shi, S. L., Lu, Y. & Wang, D. M. Experimental study on the influence of rock pore structure on pressure stimulated voltage variations based on nuclear magnetic resonance. *Eng. Geol.* **341**, 107736 (2024).

[26] Daigle, H. & Johnson, A. Combining mercury ntrusion and nuclear magnetic resonance measurements using percolation theory. *Transp. Porous Media*. **111**, 669–679 (2016).

[27] Zhang, N. et al. Coupled effects of acid and temperature on the damage characteristics of sandstone. *Rock Mech. Rock Eng.* **56**, 7839–7859 (2023).

[28] Zhu, D. Q., Zhu, S. J. & Tang, A. P. Effect of clinoptilolite on properties and fractal characteristic of pore structure of polypropylene fiber-reinforced cement-based composite. *Constr. Build. Mater.* **402**, 950618 (2023).

[29] Cao, D. P., Hou, S. & Hou, Z. Y. Constructing three-dimension digital rock with porosity information constraint: A double-network-cycled style-based deep-learning approach. *Comput. Geosci.* **193**, 105741 (2024).

[30] Cheng, G. J. et al. A multi-scale fractal model of gas flow considering the evolution of kerogen microstructure and the multi-physical coupling. *Comput. Geotech.* **165**, 105873 (2024).

[31] Yan, B. Q. et al. Fracture propagation and permeability evolution mechanism of jointed rock mass in coastal mines. *Rock Mech. Rock Eng.* **56**, 2763–2778 (2023).

[32] Cai, X., Zhou, Z. L., Tan, L. H., Zang, H. Z. & Song, Z. Y. Fracture behavior and damage mechanisms of sandstone subjected to dry-wet cycles. *Eng. Fract. Mech.* **234**, 107–109 (2020).

[33] Dong, C. L., Zhao, Y. X., Li, Z. H., He, X. S. & Yang, D. H. A multifractal geometric model for estimating spontaneous imbibition in an unsaturated fractured core-scale network in a low-permeability reservoir. *Comput. Geotech.* **176**, 106746 (2024).

[34] Liu, H. X., Ye, D. Y., Bie, P. F. & Zhu, X. Experimental study on micro-mesoscopic damage characteristics of limestone during cyclic loading and unloading. *Rock Soil Mech* **45**(3), 685–696 (2024).

[35] Sun, W. J. et al. Quantitative study of the failure characteristics of sandstone with freeze-thaw damage: Insight into the cracking behavior. *Rock Mech. Rock Eng.* **57**, 5843–5862 (2024).

[36] Zeng, T., Fang, Y., Nan, Y. L. & Yao, Y. Study on pore structure characteristics of typical shale in Yanchang formation and Longmaxi formation. *J. Eng. Geol.* **32**(04), 1176–1185 (2024a).

[37] Quan, Y. Z. et al. Cyclic wetting-drying driven geological gene changes and multi-scale degradation mechanisms of the Neogene Red-Bed sandstone in the Guide Basin, NE Tibetan Plateau. *Rock Mech. Rock Eng.* **58**, 11801–11817 (2025).

[38] Shen, J., Liu, J. P., Li, X. S. & Wang, Y. M. Mechanical degradation and failure of fractured rock mass under dry-wet-freeze-thaw cycles. *Geotech. Geol. Eng.* **44**, 74 (2026).

[39] Dong, Z. J. et al. Physicochemical deterioration mechanism of red-bed mudstone during water-rock interactions: Insights from soaking experiments. *Can. Geotech. J.* **62**, 1–18 (2025).

[40] Lou, C. D. et al. Water-rock interaction-induced degradation of Jinping marble in in-situ environments: A multi-scale analysis of mechanical behavior. *Inter J Rock Mech Min Sci* **186**, 10611 (2025).

[41] Zhao, Y. S. et al. Influence of weak interfaces on secondary crack development in crystalline rocks with varying strengths and grain sizes. *Geotech. Geol. Eng.* **44**, 90 (2024).

